# Supplementary material for: The most polyphagous insect herbivore? Host plant associations of the Meadow spittlebug, Philaenus spumarius (L.)
Source: PLoS One. 2023 Oct 4;18(10):e0291734. doi: 10.1371/journal.pone.0291734 (PMC10602594; doi:10.1371/journal.pone.0291734)

PLOS ONE

The most polyphagous insect herbivore? Host plant associations of the Meadow spittlebug, Philaenus spumarius (L.)

--Manuscript Draft--

**Manuscript Number:**	PONE-D-23-20831
**Article Type:**	Research Article
**Full Title:**	The most polyphagous insect herbivore? Host plant associations of the Meadow spittlebug, Philaenus spumarius (L.)
**Short Title:**	Host plants of Philaenus spumarius
**Corresponding Author:**	Alan J A Stewart, Ph.D.<br>University of Sussex<br>Brighton, East Sussex UNITED KINGDOM
**Keywords:**	Insect herbivore; polyphagy; host plant; Philaenus spumarius; spittlebug; citizen science
**Abstract:**	The spittlebug Philaenus spumarius (L.) feeds on 1311 host plants in 631 genera and 117 families. This appears, by a large margin, to be a host species record for herbivorous insects. The Asteraceae (222 species) and Rosaceae (110) together account for 25% of all host species. The Fabaceae (76) and Poaceae (73), are nearly tied for third and fourth place and these four families, combined with the Lamiaceae (62), Apiaceae (50), Brassicaceae (43) and Caprifoliaceae (34), comprise about half of all host species. Hosts are concentrated among herbaceous dicots but range from ferns and grasses to shrubs and trees. Philaenus spumarius is an “extreme polyphage”, which appears to have evolved from a monophage ancestor in the past 3.7 to 7.9 million years. It is also the primary European vector of the emerging plant pathogen Xylella fastidiosa. Its vast host range suggests that it has the potential to spread X. fastidiosa among multiple hosts in any environment in which both the spittlebug and bacterium are present. Fully 47.9% of all known hosts were captured in the Xylella-inspired BRIGIT citizen science P. spumarius host survey, including 358 hosts new to the documentary record, 27.3% of the 1311 total. This is a strong demonstration of the power of organized amateur observers to contribute to scientific knowledge.
**Order of Authors:**	Vinton Thompson, Ph.D.
	Claire Harkin, Ph.D.
	Alan J A Stewart, Ph.D.
**Additional Information:**	
**Question**	**Response**
**Financial Disclosure**<br><br>Enter a financial disclosure statement that describes the sources of funding for the work included in this submission. Review the submission guidelines for detailed requirements. View published research articles from *PLOS ONE* for specific examples.<br><br>This statement is required for submission and **will appear in the published article** if the submission is accepted. Please make sure it is accurate.	The BRIGIT project was funded by UK Research and Innovation (https://www.ukri.org/) through the Strategic Priorities Fund, by a grant from the Biotechnology and Biological Sciences Research Council (https://www.ukri.org/councils/bbsrc/), with support from the UK Department for Environment, Food and Rural Affairs and the Scottish Government to A.J.A.S and C.H. The funders had no role in study design, data collection and analysis, decision to publish, or preparation of the manuscript.

Powered by Editorial Manager® and ProduXion Manager® from Aries Systems Corporation

**Unfunded studies**<br>Enter: *The author(s) received no specific funding for this work.*<br><br>**Funded studies**<br>Enter a statement with the following details:<br>• Initials of the authors who received each award<br>• Grant numbers awarded to each author<br>• The full name of each funder<br>• URL of each funder website<br>• Did the sponsors or funders play any role in the study design, data collection and analysis, decision to publish, or preparation of the manuscript?<br>• **NO** - Include this sentence at the end of your statement: *The funders had no role in study design, data collection and analysis, decision to publish, or preparation of the manuscript.*<br>• **YES** - Specify the role(s) played.<br><br>* typeset	
**Competing Interests**<br><br>Use the instructions below to enter a competing interest statement for this submission. On behalf of all authors, disclose any competing interests that could be perceived to bias this work—acknowledging all financial support and any other relevant financial or non-financial competing interests.<br><br>This statement is required for submission and **will appear in the published article** if the submission is accepted. Please make sure it is accurate and that any funding sources listed in your Funding Information later in the submission form are also declared in your Financial Disclosure statement.<br><br>View published research articles from *PLOS ONE* for specific examples.	The authors have declared that no competing interests exist.

Powered by Editorial Manager® and ProduXion Manager® from Aries Systems Corporation

**NO authors have competing interests**<br><br>Enter: *The authors have declared that no competing interests exist.*<br><br>**Authors with competing interests**<br><br>Enter competing interest details beginning with this statement:<br><br>*I have read the journal's policy and the authors of this manuscript have the following competing interests: [insert competing interests here]*<br><br>* typeset	
**Ethics Statement**<br><br>Enter an ethics statement for this submission. This statement is required if the study involved:<br><br>• Human participants<br>• Human specimens or tissue<br>• Vertebrate animals or cephalopods<br>• Vertebrate embryos or tissues<br>• Field research<br><br>Write "N/A" if the submission does not require an ethics statement.<br><br>General guidance is provided below. Consult the submission guidelines for detailed instructions. **Make sure that all information entered here is included in the Methods section of the manuscript.**	N/A

Powered by Editorial Manager® and ProduXion Manager® from Aries Systems Corporation

**Format for specific study types**<br><br>**Human Subject Research (involving human participants and/or tissue)**<br>• Give the name of the institutional review board or ethics committee that approved the study<br>• Include the approval number and/or a statement indicating approval of this research<br>• Indicate the form of consent obtained (written/oral) or the reason that consent was not obtained (e.g. the data were analyzed anonymously)<br><br>**Animal Research (involving vertebrate animals, embryos or tissues)**<br>• Provide the name of the Institutional Animal Care and Use Committee (IACUC) or other relevant ethics board that reviewed the study protocol, and indicate whether they approved this research or granted a formal waiver of ethical approval<br>• Include an approval number if one was obtained<br>• If the study involved *non-human primates*, add *additional details* about animal welfare and steps taken to ameliorate suffering<br>• If anesthesia, euthanasia, or any kind of animal sacrifice is part of the study, include briefly which substances and/or methods were applied<br><br>**Field Research**<br><br>Include the following details if this study involves the collection of plant, animal, or other materials from a natural setting:<br>• Field permit number<br>• Name of the institution or relevant body that granted permission	
**Data Availability**<br><br>Authors are required to make all data underlying the findings described fully available, without restriction, and from the time of publication. PLOS allows rare exceptions to address legal and ethical concerns. See the PLOS Data Policy and FAQ for detailed information.	Yes - all data are fully available without restriction

A Data Availability Statement describing where the data can be found is required at submission. Your answers to this question constitute the Data Availability Statement and **will be published in the article**, if accepted.<br><br>**Important:** Stating 'data available on request from the author' is not sufficient. If your data are only available upon request, select 'No' for the first question and explain your exceptional situation in the text box.<br><br>Do the authors confirm that all data underlying the findings described in their manuscript are fully available without restriction?	
**Describe where the data may be found in full sentences. If you are copying our sample text, replace any instances of XXX with the appropriate details.**<br><br>• If the data are **held or will be held in a public repository**, include URLs, accession numbers or DOIs. If this information will only be available after acceptance, indicate this by ticking the box below. For example: *All XXX files are available from the XXX database (accession number(s) XXX, XXX.)*.<br>• If the data are all contained **within the manuscript and/or Supporting Information files**, enter the following: *All relevant data are within the manuscript and its Supporting Information files.*<br>• If neither of these applies but you are able to provide **details of access elsewhere**, with or without limitations, please do so. For example:<br><br>*Data cannot be shared publicly because of [XXX]. Data are available from the XXX Institutional Data Access / Ethics Committee (contact via XXX) for researchers who meet the criteria for access to confidential data.*<br><br>*The data underlying the results presented in the study are available from (include the name of the third party*	All relevant data are within the manuscript and its Supporting Information files.

and contact information or URL).<br>• This text is appropriate if the data are owned by a third party and authors do not have permission to share the data.<br><br>* typeset	
Additional data availability information:	

Powered by Editorial Manager® and ProduXion Manager® from Aries Systems Corporation

Manuscript
Click here to access/download;Manuscript;Most polyphagous insect - submitted ms.docx

The most polyphagous insect herbivore? Host plant associations of the Meadow spittlebug, *Philaenus spumarius* (L.)

Vinton Thompson[1*], Claire Harkin[2,3*], Alan J. A. Stewart[2*]

[1]Division of Invertebrate Zoology, American Museum of Natural History, New York, New York, United States of America

[2]School of Life Sciences, University of Sussex, Falmer, Brighton, East Sussex, United Kingdom

[3]Ancient Tree Forum, London, United Kingdom

*Corresponding authors

E-mail: vthompson@mcny.edu (VT), claire.harkin@ancienttreeforum.org.uk (CH), a.j.a.stewart@sussex.ac.uk (AJAS)

Short title: Host plants of *Philaenus spumarius*

ORCID numbers:

VT: 0000-0003-3257-0107; CH: 0000-0002-4106-038X; AJAS: 0000-0001-7878-8879

Abstract

The spittlebug *Philaenus spumarius* (L.) feeds on 1311 host plants in 631 genera and 117 families. This appears, by a large margin, to be a host species record for herbivorous insects. The Asteraceae (222 species) and Rosaceae (110) together account for 25% of all host species. The Fabaceae (76) and Poaceae (73), are nearly tied for third and fourth ~~place and these~~ four families, combined with the Lamiaceae (62), Apiaceae (50), Brassicaceae (43) and Caprifoliaceae (34), comprise about half of all host species. Hosts are concentrated among herbaceous dicots but range from ferns and grasses to shrubs and trees. *Philaenus spumarius* is an "extreme polyphage", which appears to have evolved from a monophage ancestor in the past 3.7 to 7.9 million years. It is also the primary European vector of the emerging plant pathogen *Xylella fastidiosa*. Its vast host range suggests that ~~it~~ has the potential to spread *X. fastidiosa* among multiple hosts in any environment in which both the spittlebug and bacterium are present. Fully 47.9% of all known hosts were captured in the *Xylella*-inspired BRIGIT citizen science *P. spumarius* host survey, including 358 hosts new to the documentary record, 27.3% of the 1311 total. This is a strong demonstration of the power of organized amateur observers to contribute to scientific knowledge.

Introduction

The Meadow spittlebug *Philaenus spumarius* (L.) (Hemiptera: Aphrophoridae) is one of the world's most widespread and abundant insects (Fig 1). It is also the major European vector of *Xylella fastidiosa* Wells et al., an emerging bacterial plant pathogen that threatens crops as diverse as grapes, almonds, citrus and olives [1–4]. Despite its importance, the last comprehensive review of *P. spumarius* host plants is almost 70 years old [5]. Recent concern with *X. fastidiosa* has led to a proliferation of new studies on *P. spumarius* hosts (e.g. [6–11]), including the BRIGIT citizen scientist initiative in Britain that enlisted amateurs to identify *P. spumarius* host plants [12].

Fig 1. The meadow spittlebug *Philaenus spumarius* (L.). a) Frothy spittle mass on one of its common host plants, the legume *Lotus corniculatus*, bird's-foot trefoil. The nymph is hidden within. b) Spittle foam removed to expose nymph. The red dot is its right eye. Note the swollen sucking pump anterior to the eyes. c) Adult *P. spumarius*. This is the most common of several colour forms. Photographs by Claire Harkin.

The present work has multiple aims. First, we bring together disparate sources in a publicly available, comprehensive, documented compilation of *P. spumarius* hosts. This will be of use to investigators studying the spread of *X. fastidiosa* and broader biological phenomena, such as the evolution of xylem feeding insects and the evolution of extreme polyphagy. Second, we analyse the broad patterns of *P. spumarius* host exploitation, place it in the context of other highly polyphagous insects, and examine the implications of host patterns for *X. fastidiosa* spread. Third, we suggest some useful guidelines for future surveys of spittlebug host plants. Finally, we assess the efficacy of the BRIGIT project in corroborating existing host records and establishing

new ones. We believe this is the first time a citizen science project of this scope has been tested against the historical record.

Philaenus spumarius first became a major subject of study in the late 1940s and early 1950s in the United States and Canada for its role as a serious non-native pest of alfalfa (*Medicago sativa*) and other forage legumes [5]. From about 1960 to 2010 it was primarily of interest to entomologists and ecologists working on insect population dynamics and energetics [13–16] and to researchers interested in its remarkable colour polymorphism [17–20]. More recently, it has attracted considerable attention from applied entomologists and pest management scientists, after it was shown to be the primary European vector of *X. fastidiosa*, a pathogen that causes Olive Quick Decline Syndrome (OQDS), which has devastated olive groves in the Apulia region of Italy [1,21]. This threat, along with the recent association of *P. spumarius* with Almond leaf scorch disease (ALSD) in the Alicante and Balearic Islands regions of Spain [22,23], has generated intense research activity focused on ~~*P. spumarius*,~~ reflected in an exponential recent rise in publications and citations (175 papers and 2,699 citations since 2010; Web of Science, accessed 21 May 2023). *Xylella fastidiosa* was introduced from the New World to Europe relatively recently [23]. It has now been detected in over 500 plant species, including crops, ornamentals and trees, across many plant families [24]. To predict which agricultural and natural plant species and ecosystems are at risk, it is crucial to understand the host range of the vector.

Information on *P. spumarius* host plants is scattered across many languages and continents, from the primary scientific literature to amateur entomology publications, agricultural tracts and unpublished sources. Schmidt [25,26], apparently an amateur, published the earliest extensive

host lists in 1914, totaling 137 species, in a German civic booster journal promoting the progress of science and industry. The next major contributions came from Hawaii [27,28], following the introduction and proliferation of *P. spumarius* on Big Island in the 1940s. This work was followed shortly by the 1950 publication of 97 California hosts observed by DeLong and Severin [29] in the course of the first experiments demonstrating that *P. spumarius* and other spittlebugs can transmit what is now known to be *X. fastidiosa*. A series of host lists in American PhD theses [30–33] culminated in 1954 in the 383 species compilation of Weaver & King [5]. Metcalf's encyclopedic catalog of the Aphrophoridae [34] includes annotated references to host information in *P. spumarius* publications through 1955, a very useful source of otherwise obscure observations. In 1965 Noury [35] compiled a list of about 167 hosts from France and Middle and Northern Europe. Halkka et al. [36] provide a list of 165 hosts based on 1960s field observations in Finland. A 1993 PhD thesis by Booth [37] includes records of 90 hosts in natural areas in Wales and New Zealand. Jennifer Owen [38] and Denis Owen [39] record observing 143 host species in 47 plant families in their late twentieth century English suburban garden, detailed in notes that we recovered from J. Owen's papers in the Leicester Museum & Art Gallery. More recently, concern over *X. fastidiosa* has stimulated a golden age of *P. spumarius* host studies in the Mediterranean region (references below). We have collected, evaluated and distilled these sources and all others available to us into a single, readily accessible summary table in searchable format.

Methods

Sources of information

This work is based on the published literature, both formal and informal, host records associated with museum specimens, personal observations, private communications from colleagues, and the BRIGIT citizen science effort carried out in Britain. The first four sources are encompassed in an unpublished world database of spittlebug host plants built and maintained by VT. ~~With no little irony, it~~ contains most published host records for most spittlebugs (Hemiptera: Cercopoidea) *except* for *P. spumarius*. *Philaenus spumarius* was initially exempted from full incorporation because the number of host records was enormous and because there seemed to be little point in recording the hosts of an insect that appeared to feed on most any available plant. That changed with increasing concern about the role of *P. spumarius* as a *X. fastidiosa* vector. Thousands of *P. spumarius* records have been added, increasing the database from about 5,000 to 9,000 records over the past two years. Even with these additions it is not comprehensive for *P. spumarius*, missing for example, all of the BRIGIT citizen science records reported here, which are in a separate database maintained by CH and AJAS. The hosts presented here include all known to us as of May 2023.

The BRIGIT project [12] ran from 2019 to 2021 with the aim of improving surveillance and response capacity for *X. fastidiosa* should it be introduced into the UK. A key objective of the project was to develop a greater understanding of the distribution and host plant preferences of *P. spumarius*, identified as the primary vector of the bacterium in Europe [40]. Citizen scientists were encouraged to submit sightings via a national website for natural history observations or a bespoke portal, supported by identification aids on the BRIGIT website.

Sources of ambiguity

Anomalies in Weaver & King

Prior to this work, Weaver & King's 1954 compilation [5] has been the primary source of *P. spumarius* host records. It originated as a shorter host list in a preceding thesis by King [30]. Ostensibly, it is an amalgam of 26 cited sources, combined with personal observations in Ohio by Weaver and King themselves. We have tracked down and examined all of the original sources cited~~, some of them quite obscure~~. This revealed several anomalies. For example, Weaver and King state that all the records in their compilation are for nymphal hosts and go on to say that they omitted the long 1950 California list compiled by DeLong & Severin [29] because those authors did not distinguish between nymphal and adult hosts. Nevertheless, they still cite four of the 97 hosts reported in that work, while omitting the rest. They list hosts from Osborn's 1916 work [41] without reservation, though Osborn too omits information on whether his observations are for nymphs or adults. Unlike Osborn, DeLong & Severin provide dates and locales for their observations. Given patterns of local climate in California (with which VT is familiar), it may be reasonably inferred that they observed nymphs for all listed hosts.

Weaver and King also inexplicably omit about two dozen nymphal hosts listed in Teller [32], while incorporating 33 others. Likewise, their treatment of Marshall [33] is problematic. They include most of his records, despite the fact that he does not specify life stage, but attribute one host record to him that is not in his work (*Rumex acetosa*). They also attribute two of Marshall's records (*Pinus strobus* and *Rumex occidentalis*) to Krauss [42], making it appear that they came from Hawaii rather than New York State. In another oddity, they misattribute a work by Davis [27] to the authors of a preceding work by Davis & Mitchell [28] and get the title of the paper by Davis wrong. These mistakes have the look of clerical errors. In the case of Licent [43], they

include three of the hosts listed, but omit three others, perhaps a case of oversight, since the three unlisted hosts are in a different section of a very long treatise.

More disconcertingly, and for no obvious reason, Weaver and King omit 39 hosts out of 85 listed in Schmidt's second 1914 paper [25] and misstate the date of that publication as 1915. Given these anomalies and inconsistencies, we have included only hosts that could be corroborated in the original cited works and incorporate, where appropriate, hosts listed in the original sources that Weaver and King omitted. We include the host records Weaver and King report as their own observations.

Sitting records versus host records

We distinguish among records for nymphal hosts, adult hosts and plants that host both stages. Nymphal host records are unambiguous because nymphs (Figure 1b) cannot produce spittles (Figure 1a) without feeding. Adult records are more problematic. Adult *P. spumarius* (Figure 1c) are active insects and move about from plant to plant. Unless accompanied by direct observation of feeding in the form of regularly expelled droplets of excreta, adult records may represent "sitting" rather than feeding individuals. This produces inevitable "noise" in adult host records. To minimize this noise, we have omitted instances in which adult host records are clearly problematic, including those based on single collected specimens. Nevertheless, some of our adult host records are likely in error, or, at a minimum, insecurely documented. On the other hand, there is no doubt that adults do feed on a wide variety of plants. Aside from eggs, *P. spumarius* does not have an inactive or dormant stage. They must feed to live, and feed in quantity. When there are a lot of adults on a particular plant or when adults associate with multiple individuals of the same plant species, chances are they are feeding. This is particularly

true of the many adults found on trees and shrubs in dry climate summers, when plants in the herbaceous understory have browned out for the season [22,44,45].

Spittlebug identity in the BRIGIT citizen science data

The citizen science survey benefitted from the specific character of the British spittlebug fauna. In many other areas there are common spittlebug species frequenting herbaceous dicots that might easily be misidentified for *P. spumarius* based on observation of spittles alone. This is less the case in Britain, but two British genera, *Neophilaenus* and *Aphrophora*, do include spittlebugs that might be mistaken in the spittle stage for *P. spumarius* if the nymphs themselves are not examined carefully. The four British *Neophilaenus* species are restricted almost entirely to monocots and can be screened out by eliminating ambiguous records from grasses and sedges. The four *Aphrophora* species are primarily insects of trees and shrubs, but the nymphs of the most common and widespread species, *Aphrophora alni* Fallén, also occasionally feed on herbaceous plants. Thus, although the nymphs are distinct, identification based solely on spittles runs the risk of conflating *P. spumarius* and *A. alni*. In practice, however, extensive field collections across the UK undertaken during the BRIGIT project found very few *A. alni* on herbaceous plants; consequently, this risk is judged to be minimal.

Validation of BRIGIT citizen science host plant records adopted a highly conservative approach: 1) all records based on adult *P. spumarius* were rejected due to the difficulty in distinguishing between sitting and feeding behaviours, as previously described; 2) nymph host records were accepted only where *P. spumarius* identification could be confirmed via accompanying photographs; 3) host records based on the presence of spittle were attributed to *P. spumarius* only when the reported host was an herbaceous dicot.

Changing plant taxonomy

Botanical nomenclature poses additional hurdles. Some of our sources are over 100 years old and plant taxonomy has moved on. Wherever possible, we have updated species names to conform with current usage. Where we encountered nomenclatural ambiguity, we used two sources to determine currently valid names: the Integrated Taxonomic Information System [46] and Kew Plants of the World Online [47]. We have updated plant family-level taxonomy to be consistent with contemporary usage, following the template of Christenhusz & Byng [48]. This pared down the number of families by about 9%. In many cases, particularly those involving agricultural or popular sources, we had to make reasonable inferences from common names in multiple languages, an endeavor with its own hazards. We are confident that most of our botanical names are correct and up to date, but in a work of this scale, from such diverse sources, some errors are inevitable. We also recognize the possibility that some of the original plant identifications may have been in error, another, hopefully sma[illegible]ource of noise in the data.

Criteria for inclusion of records in this compilation

We have included all naturally occurring hosts for which we have found at least one usable record. We exclude records based solely on laboratory experiments. Most records are reported to species level. In instances in which we encountered a mix of records that were generic-only and species-specific for a given genus, we include only species-specific records, to avoid the possibility of double counting. This makes our host number estimates more conservative, as, in all likelihood, some of the excluded generic records represent species distinct from but

congeneric to those included. Holopainen & Varis [49] report that using this criterion decreased their host total for *Lygus rugulipennis* from 437 to 402, a reduction of 8%. Application of the same criteria to underlying data for the scales *Aspidiotis nerii* and *Hemiberlesia lataninae* [50] reduced the number of species records by about 12.5% in each case. As noted above, we also exclude records of adult hosts based on single specimens.

In cases in which we have multiple records for the same host species, we have given preference in the following order: formal scientific publications (journal articles, books, stand-alone scientific publications), followed by theses and dissertations, followed by more ephemeral Internet sources, followed by unpublished observations by cited observers. Where we have records for multiple geographic areas, we list all areas and cite at least one record for each. For hosts with records for both nymphs and adults, we cite at least one record for each stage. The goal is to keep the list of references for each host species compact, while documenting the known geographical distribution and observed stages for each host. The 358 citizen science host observations new to science are marked ●●●, while those new to the UK are marked ●●, and those confirming earlier local observations are marked ●.

The geographic units chosen for this report include several with natural boundaries. New Zealand (NZ) and Hawaii (HI) represent discrete island areas where *P. spumarius* has been introduced. The Azores (AZ), where *P. spumarius* is likely but not certainly recently introduced [51–53] are a special island case, but one with very few host records. In North America, where *P. spumarius* is introduced, it has a disjunct distribution [54], divided by the dryer midsection of the continent into discrete eastern (ENA) and western areas (WNA). Britain and Ireland (B&I) represent another natural division. We somewhat arbitrarily divide continental Europe into four areas: Finland and Scandinavia (F&S), Western Europe (WE), Eastern Europe (EE), and the

Mediterranean Basin (MED). See the footnotes to Table 1 for the detailed boundaries. A number of Noury's [35] listings are from a source covering "Middle and Northern Europe", a category that does not fit our arbitrary divisions. These records are recorded as M&NE. A few North American references do not specify which section of the continent. These are recorded as EorWNA. Although *P. spumarius* is frequent in Kyrgyzstan [55] and is reported across Asia to China and Japan (refs. in [34]), we found only one host record east of European Russia, in Uzbekistan [56]. *P. spumarius* has been reported once, on strawberries, on the island of Réunion in the Indian Ocean [57] but, in the absence of later reports, seems not to have become established.

Results

Table 1 lists 1311 species of *Philaenus spumarius* host plants, by plant family and binomial in alphabetical order within families. It also ~~gives~~ the life stages observed (nymph and/or adult), the geographical area(s) in which the host association was observed, the BRIGIT citizen science observation status (if any), and selected references.

[the narrative text resumes after Table 1]

Table 1. *Philaenus spumarius* host plants, by plant family, life stage, geographic occurrence, and BRIGIT project status. See footnotes for a key to and explanation of abbreviations.

Host species by family	Stage	Geographic area(s)	Cit. sci. obs.	Selected record source(s)
**Acanthaceae**				
Acanthus mollis	N	B&I	●●●	
**Adoxaceae**				
Sambucus canadensis	N	ENA		[58]
Sambucus nigra	N	B&I WE WNA	●●	[25,26,29]
Sambucus racemosa	N	F&S WE		[35,36]
**Aizoaceae**				
Carpobrotus chilensis	N	B&I	●●●	
Carpobrotus edulis	N,A	B&I MED WNA	●●	[59,60]
Cleretum bellidiforme	N	B&I	●●●	
Sesuvium portulacastrum	N	HI		[27]
**Alismataceae**				
Alisma triviale	N	ENA		[58]
**Alstroemeriaceae**				
Alstroemeria sp.	N	B&I		HMBL-England
**Altingiaceae**				
Liquidambar styraciflua	N	B&I	●●●	

**Amaranthaceae**				
Alternanthera dentata	N	B&I	●●●	
Amaranthus retroflexus	A	EE		[61]
Atriplex prostrata	A	WNA		VT-CA-2013-A
Beta vulgaris	N	B&I MED ENA HI	●●	[5,27,62]
Chenopodium album	N,A	F&S B&I WE MED ENA	●	[5,35,36,63,64]
Spinacia oleracea	N	B&I M&NE EorWNA	●●	[35,65]
**Amaryllidaceae**				
Allium cepa	N	B&I ENA	●●	[5]
Allium sativum	N	B&I	●●●	
Allium schoenoprasum	N	F&S B&I	●●	[36]
Allium schubertii	N	B&I	●●●	
Allium siculum	N	B&I	●●●	
Allium sphaerocephalon	N	B&I	●●●	
Allium triquetrum	N	B&I	●●●	
Allium vineale	N	ENA		[32]
Allium × proliferum	N	B&I		J. Owen
Narcissus sp.	A	ENA		[66]
**Anacardiaceae**				
Cotinus coggygria	N	B&I WE	●●	[67]
Pistacia lentiscus	N,A	MED		[68,69]
Pistacia terebinthus	A	MED		[68]
Rhus glabra	N	ENA		[32]

Toxicodendron diversilobum	N	WNA		[29]
Toxicodendron radicans	N	ENA		[32]
**Apiaceae**				
Aciphylla sp.	?	NZ		[70]
Aegopodium podagraria	N	F&S B&I WE	●●	[25,26,36]
Ammi majus	N	B&I	●●●	
Ammi visnaga	N	MED		[71]
Anethum graveolens	N	B&I	●●●	
Angelica archangelica	N	F&S		[36]
Angelica sylvestris	N	F&S B&I	●●	[36]
Anthriscus cerefolium	N	B&I		J. Owen
Anthriscus sylvestris	N	F&S B&I WE MED	●	[9,36,63,72]
Anthriscus vulgaris	N	WE		[73]
Apium graveolens	N	B&I MED ENA WNA HI	●●	[5,29,42,74]
Apium nodiflorum	N	B&I	●●●	
Astrantia major	N	B&I	●●●	
Carum carvi	N	WE		[26]
Caucalis platycarpos	N	MED		[75]
Cervaria sp.	N	MED		[10]
Chaerophyllum bulbosum	N	M&NE		[35]
Chaerophyllum hirsuitum	N	M&NE		[35]
Chaerophyllum temulum	N	WE		[35]
Conium maculatum	N	WNA		[29]
Conopodium majus	N	B&I		[15,63]

Coriandrum sativum	N	B&I	●●●	
Crithmum maritimum	N	B&I	●●●	
Daucus carota	N,A	F&S B&I MED ENA WNA HI NZ	●●	[5,7,28,29,36,37,76]
Eryngium campestre	N	MED		[77]
Eryngium maritimum	N	B&I	●●●	
Eryngium planum	N	B&I	●●●	
Falcaria vulgaris	N	WE		[25,26]
Foeniculum vulgare	N	B&I MED WNA	●●	[9,10,60,78] VT-CA-2021-N
Heracleum mantegazzianum	N	B&I	●●●	
Heracleum maximum	N,A	WNA		[79] VT-WA-2003-A
Heracleum sphondylium	N	B&I WE	●	[25,26,37]
Laserpitium latifolium	N	M&NE		[35]
Levisticum officinale	N	B&I WE ENA	●●	[35,80]
Ligusticum scoticum	N,A	B&I		[81]
Myrrhis odorata	N	B&I	●●●	
Pastinaca sativa	N	B&I WE ENA HI	●	[5,25,26,28,63]
Petroselinum crispum	N	B&I WE ENA WNA HI	●	[5,29,37,42,82]
Peucedanum alsaticum	N	M&NE		[35]
Peucedanum officinale	N	B&I		HMBL-England
Peucedanum palustre	N	F&S		[36]
Pimpinella anisum	A	MED		[74]
Pimpinella major	N	WE		[25]
Pimpinella saxifraga	N,A	F&S B&I WE MED	●●	[35,36,74]
Sanicula liberta	N	WNA		[29]

Scandix pecten-veneris	N	MED		[6]
Seseli tortuosum	N	MED		[60]
Smyrnium olusatrum	N	B&I	●●●	
Tordylium sp.	N	MED		[10]
Torilis japonica	N	WE	●●	[25]
Torilis nodosa	N	MED		[8,60]
**Apocynaceae**				
Alyxia stellata	N	HI		[27]
Asclepias sp.	N	ENA		[32]
Trachelospermum jasminoides	N	B&I	●●●	
Vinca major	N	B&I WNA	●●	[29]
Vinca minor	N	B&I	●●●	
**Aquifoliaceae**				
Ilex anomala	N	HI		[28]
Ilex verticillata	N	B&I	●●●	
**Araceae**				
Acorus calamus	N	B&I	●●●	
Arum italicum	N	WE		[83]
Zantedeschia aethiopica	N	B&I WNA	●●	[29]
**Araliaceae**				
Hedera canariensis	N	WNA		[29]
Hedera colchica	N	B&I	●●●	
Hedera helix	N,A	B&I WNA	●	[29,37,84]

**Asparagaceae**				
Aloe vera	N	B&I	●●●	
Asparagus officinalis	A	MED		[85]
Chlorogalum pomeridianum	N	WNA		[29]
Chlorophytum laxum	N	B&I	●●●	
Convallaria majalis	N	F&S		[36]
Cordyline australis	N	B&I	●●●	
Cordyline fruticosa	N	HI		[28]
Eucomis comosa	N	B&I	●●●	
Hyacinthoides hispanica	N	B&I	●●●	
Hyacinthoides non-scripta	N	B&I	●	[37]
Maianthemum bifolium	N	F&S		[36]
Muscari comosum	N	MED		[11]
Ornithogalum ortophyllum	N	MED		[86]
Ruscus aculeatus	N	MED		[86]
**Asphodelaceae**				
Asphodelus ramosus	N	MED		[44]
Hemerocallis fulva	N	ENA		[5]
Hemerocallis lilioasphodelus	N	B&I	●●●	
Kniphofia galpinii	N	B&I	●●●	
Simethis planifolia	N	MED		[60]
**Aspleniaceae**				
Athyrium filix-femina	N	F&S B&I	●●	[36]

Thelypteris palustris	N	ENA		[87]
**Asteraceae**				
Acanthospermum australe	N	HI		[27]
Achillea ageratum	N	B&I	●●●	
Achillea filipendulina	N	B&I		J. Owen
Achillea millefolium	N	F&S B&I WE ENA WNA NZ	●	[36,37,63,87,88] VT-CA-2021-N
Achillea ptarmica	N	F&S	●●	[36]
Adenostyles alpina	N	MED		HMBL-Italy
Ambrosia artemisiifolia	N,A	EE ENA		[32,89]
Ambrosia trifida	N	ENA		[32]
Anaphalis margaritacea	N,A	ENA WNA		[87,90,91]
Andryala integrifolia	N	WE MED		[25,92]
Antennaria plantaginifolia	N	ENA		[5]
Anthemis arvensis	N	WE		[25]
Anthemis arvensis	N	MED		[7,11,69]
Anthemis chia	N	MED		[6]
Anthemis cotula	N	MED		[93]
Arctium lappa	N	WE		[25]
Arctium minus	?	ENA		[94]
Arctium tormentosum	N	F&S		[36]
Arctotheca calendula	?	MED		[95]
Argyranthemum frutescens	N	B&I WE		J. Owen [35]
Arnica montana	N	B&I	●●●	
Artemisia abrotanum	N	B&I WE	●●	[35]

Artemisia absinthium	N	F&S		[36]
Artemisia campestris	N	WE, MED		[25,60]
Artemisia caudata	N	ENA		VT-MN-2001-N
Artemisia dracunculus	N	B&I ENA	●●	[5]
Artemisia tridentata	A	WNA		VT-UT-2003-A
Artemisia verlotiorum	A	WE		[96]
Artemisia vulgaris	N	F&S B&I WE MED WNA HI	●●	[9,28,29,36,72]
Aster alpinus	N	B&I	●●●	
Aster amellus	N	B&I	●●●	
Asteriscus sp.	?	MED		[97]
Baccharis pilularis	N	WNA		[29]
Bahia ambrosioides	N	B&I	●●●	
Bellis perennis	N	B&I MED ENA	●●	[33,98]
Bidens pilosa	N	HI		[28]
Bidens tripartida	N	F&S WE		[35,36]
Brachyglottis grayi	N	B&I	●●●	
Buphthalmum salicifolium	N	B&I M&NE	●●	[35]
Calendula arvensis	N	MED		[7,11]
Calendula officinalis	N	B&I MED	●●	[75]
Carduus acanthoides	N	NZ		[37]
Carduus crispus	N	B&I	●●●	
Carduus nutans	N,A	B&I ENA		[63,99]
Carduus pycnocephalus	N,A	WNA		[100]
Carduus tenuiflorus	N	MED		[77]

Carlina hispanica	A	MED		[7]
Catananche caerulea	N	B&I	●●●	
Celmisia sp.	?	NZ		[70]
Centaurea aspera	N	B&I		HMBL-England
Centaurea centaurium	N	B&I	●●●	
Centaurea cyanus	N	B&I, WE	●●	[25]
Centaurea jacea	N	M&NE		[35]
Centaurea macrocephala	N	B&I	●●●	
Centaurea montana	N	B&I	●●●	
Centaurea nigra	N	B&I	●	[37]
Centaurea ornata	A	MED		[7]
Centaurea phrygia	N	F&S		[36]
Centaurea scabiosa	N	B&I	●●●	
Centaurea solstitialis	N,A	WNA		[101]
Chondrilla juncea	N	WE MED		[6,25,92]
Chrysanthemum carinatum	N	F&S		[36]
Chrysanthemum makinoi	N	WE		[35]
Chrysanthemum maximum	N	WNA HI		[28,29]
Chrysanthemum vulgare	N	WE		[25]
Chrysanthemum x morifolium	N	B&I	●●●	
Cicerbita alpina	N	B&I	●●●	
Cichorium intybus	N	MED ENA	●●	[11,32,75]
Cirsium arvense	N,A	F&S B&I WE MED ENA NZ	●	[7,36,37,84,87,88]
Cirsium discolor	N	ENA		[102]

Cirsium dissectum	N	B&I	●●●	
Cirsium eriophorum	N	B&I		[63]
Cirsium heterophyllum	N	F&S		[36]
Cirsium lanceolatum	N	WNA		[29]
Cirsium oleraceum	N	M&NE		[35]
Cirsium palustre	N	F&S B&I WNA	●●	[36,103]
Cirsium rivulare	N	B&I	●●●	
Cirsium vulgare	N	F&S B&I ENA WNA	●●	[5,36,103,104]
Coleostephus myconis	N	MED		[92]
Conyza sumatrensis	N	HI		[28]
Coreopsis sp.	?	?		[57]
Cosmos atrosanguineus	N	B&I	●●●	
Crepis biennis	N	B&I WE MED	●●	[11,88]
Crepis capillaris	N	B&I WE NZ	●●	[26,37]
Crepis neglecta	N	MED		[11]
Crepis paludosa	N	F&S		[36]
Crepis vesicaria	N	B&I	●●●	
Cynara cardunculus	N	B&I WE MED WNA	●●	[29,35,105]
Cynara scolymus	N	B&I	●●●	
Dahlia sp.	N	WE WNA HI		[25,28,29]
Dimorphotheca pluvialis	N	B&I	●●●	
Dittrichia viscosa	N,A	MED		[7,11,106]
Dubautia scabra	N	HI		[28]
Echinacea pallida	N	B&I	●●●	

Echinops bannaticus	N	B&I	●●●	
Echinops ritro	N	B&I	●●●	
Echinops sphaerocephalus	N	B&I	●●●	
Erigeron annuus	N	ENA NZ		[37,107]
Erigeron bonariensis	N	NZ		[37]
Erigeron canadensis	N	WE MED ENA		[5,25,60]
Erigeron glaucus	N	WNA		[108]
Erigeron karvinskianus	N	B&I	●●●	
Erigeron philadelphicus	A	WNA?		UCRC
Erigeron strigosus	N	ENA		[32]
Erigeron sumatrensis	N	B&I HI	●●	[28]
Eupatorium cannabinum	N	B&I		CH-England-2019-N
Eurybia divaricata	N	B&I	●●●	
Euthenia graminifolia	N	ENA		VT-MN-2001-N
Eutrochium maculatum	N	B&I	●●●	
Felicia petiolata	N	WNA		[29]
Galactites tomentosa	N	MED		[8]
Galinsoga ciliata	A	ENA		[109]
Galinsoga parviflora	A	ENA		[109]
Gamochaeta purpurea	N	HI		[28]
Gazania rigens	N	WNA		[29]
Glebionis coronaria	N	F&S MED		[11,36]
Gnaphalium sylvaticum	N	M&NE		[35]
Hedypnois cretica	N	MED		[11]

Helianthus annuus	N	B&I	●●●	
Helianthus tuberosus	N	B&I	●●●	
Helicanthus giganteus	N	B&I	●●●	
Helichrysum italicum	N	B&I MED	●●	[60]
Heliopsis scabra	N	WE		[35]
Helminthoteca echioides	N	B&I MED WNA		[6,7,9,110]
Hemizonia congesta .	?	WNA		[111]
Hieracium caespitosum	N,A	F&S ENA NZ		[36,70,87,112]
Hieracium lepidulum	N,A	NZ		[70]
Hieracium maculatum	N	B&I	●●●	
Hieracium murorum	N	MED		HMBL-Italy
Hieracium pilosella	N,A	NZ		[70]
Hieracium praealtum	N	NZ		[112]
Hieracium tridentatum	N	WE		[25]
Hieracium umbellatum	N	F&S		[36]
Hyoseris sp.	N	MED		[10]
Hypochaeris glabra	N	MED,WNA		[29,92]
Hypochaeris radicata	N,A	B&I MED ENA WNA HI	●	[5,28,37,40] SEMC
Hypochoeris maculata	N	F&S		[36]
Inula sp.	N	MED		[10]
Jacobaea aquatica	N	B&I	●●●	
Jacobaea maritima	N	B&I	●●●	
Jacobaea paludosa	N	B&I	●●●	
Jacobaea vulgaris	N	B&I	●●●	

Lactuca muralis	N	B&I	●●●	
Lactuca sativa	N	B&I EorWNA HI	●●	[28,65]
Lactuca serriola	N	B&I ENA	●●	[104]
Lactuca virosa	N	ENA		[5]
Lapsana communis	N	B&I WE	●●	[25,26]
Leontodon hispidus	N	B&I WE	●●	[26]
Leontodon taraxacoides	N	NZ		[37]
Leucanthemum merinoi	N	MED		[60]
Leucanthemum vulgare	N	B&I WE ENA	●●	[32,35]
Leucanthemum x superbum	N	B&I	●●●	
Leucophyta brownii	N	B&I	●●●	
Logfia gallica	N	MED		[92]
Madia elegans	N	WNA		[29]
Matricaria camomilla	N	WE		[82]
Matricaria discoidea .	N	WE		[35]
Oclemena acuminata	N	ENA		[87]
Oclemena nemoralis	N	ENA		[87]
Onopordum acanthium	A	MED		[7]
Ozothamnus rosmarinifolius	N	B&I		HMBL-England
Pallenis spinosa	N	MED		[11]
Petasites hybridus	N	B&I WE	●●	[25]
Petasites pyrenaicus	N	B&I WE	●●	[35]
Picris hieracioides	N	MED		[11]
Pilosella aurantiaca	N	B&I ENA	●●	[87]

Pilosella caespitosa	N,A	B&I ENA NZ	●●	[33,70]
Pilosella officinarum	N	B&I	●●●	
Pluchea odorata	N	HI		[28]
Psephellus dealbatus	N	B&I	●●●	
Pulicaria dysenterica	N	B&I	●●●	
Reichardia sp.	N	MED		[10,78]
Rhaponticum carthamoides	N,A	WE		[113]
Rhaponticum scariosum	N	B&I	●●●	
Rudbeckia fulgida	N	B&I	●●●	
Rudbeckia hirta	N	F&S B&I	●●	[114]
Rudbeckia laciniata	N	WNA	●●	[29]
Santolina chamaecyparissus	N	B&I	●●	[115]
Scolymus hispanicus	N	MED		[77,116]
Scorzonera aristata	N	MED		[11]
Scorzoneroides autumnalis	N	F&S B&I WE	●●	[26,36]
Senecio jacobaea	N	B&I		[37,63]
Senecio mikanioides	N	HI		[28]
Senecio squalidus	N	B&I	●●●	
Senecio vulgaris	N	MED		[60,69]
Silybum marianum	N	B&I MED	●●	[6]
Solidago altissima	N,A	WE ENA HI		[28,102,117,118]
Solidago canadensis	N	F&S B&I	●●	[36]
Solidago gigantea	N	WE ENA		VT-MN-2001-N
Solidago rugosa	N	ENA		[87]

Solidago sempervirens	N	ENA		[87]
Solidago shortii	A	ENA		[119]
Solidago virgaurea	N	F&S B&I WE	●●	[35,36]
Sonchus arvensis	N	B&I MED	●●	[11]
Sonchus asper	N	B&I MED WNA	●●	[9,29]
Sonchus oleraceus	N	B&I MED WNA HI NZ	●●	[6,7,9,28,29,37,69]
Sonchus terrenimus	N	MED		[92]
Stokesia laevis	N	B&I	●●●	
Symphyotrichum cordifolium	N	B&I ENA	●●	[104]
Symphyotrichum ericoides	N	ENA		[104]
Symphyotrichum lanceolatus	N	M&NE		[35]
Symphyotrichum lateriflorum	N	B&I	●●●	
Symphyotrichum novae-angliae	N	B&I	●●●	
Symphyotrichum novi-belgii	N	B&I	●●●	
Symphyotrichum tradescantii	N	B&I		[120]
Symphyotrichum turbinellum	N	B&I	●●●	
Symphytum officinale	N	B&I WE	●●	[82]
Tagetes lucida	N	B&I	●●●	
Tanacetum parthenium	N	B&I	●●●	
Tanacetum vulgare	N	F&S B&I WE WNA	●●	[29,35,36]
Taraxacum kok-saghyz	N	B&I	●●●	
Taraxacum officinale	N	B&I WE, MED ENA WNA NZ	●	[5,25,29,37,40,75,83]
Tolpis umbellata	N	MED		[11,69]
Tragopogon dubius	N	B&I	●●●	

Tragopogon porrifolius	N	WNA		[29]
Tragopogon pratensis	N	B&I	●●●	
Tripleurospermum inodorum	N	F&S B&I WE	●●	[25,36]
Tripolium pannoncium	N	B&I	●●●	
Tussilago farfara	N	F&S B&I	●●	[36]
Urospermum dalechampii	N	MED		[9,11,69]
Urospermum picroides	N	MED		[11]
Wyethia amplexicaulis	A	WNA		AMNH
**Balsaminaceae**				
Impatiens capensis	N	ENA		[87]
Impatiens glandulifera	N	B&I	●●●	
Impatiens noli-tangere	N	WE		[26]
**Berberidaceae**				
Berberis darwinii	N	B&I	●	[39]
Berberis julianae	N	B&I	●●●	
Berberis thunbergii .	N	B&I ENA	●●	[5]
Mahonia aquifolium	N	B&I	●	[39]
Nandina domestica	N	B&I	●●●	
Podophyllum peltatum	N	B&I	●●●	
**Betulaceae**				
Alnus glutinosa	N	F&S B&I	●●	[36,121]
Alnus incana	N	F&S		[36]
Alnus rubra	A	WNA		VT-OR-2003-A

Betula alleghaniensis	A	ENA		[122]
Betula nigra	N	B&I	●●●	
Betula papyrifera	A	ENA		[122]
Betula pendula	N	F&S		[36]
Betula populifolia	A	ENA		[122]
Betula pubescens	N	F&S	●●	[36,121]
Carpinus orientalis	?	MED		[97]
Corylus avellana	N,A	F&S B&I WE	●●	[67,121]
Ostrya carpinifolia	A	MED		[123]
**Boraginaceae**				
Amsinckia menziesii	N	WNA		RKpercom
Amsinckia spectabilis	N	WNA		RKpercom
Anchusa officinalis	N	WE		[26]
Asperugo procumbens	N	WE		[25]
Borago officinalis	N	B&I MED	●●	[9,116]
Echium pininana	N	B&I	●●●	
Echium plantagineum	N	MED		[7,92]
Echium vulgare	N	F&S, B&I MED	●●	[75,124]
Glandora diffusa	N	B&I	●●●	
Heliotropium arborescens	N	WNA		[29]
Lithospermum croceum	N	ENA		VT-MN-2001-N
Lithospermum officinale	N	B&I		[63]
Myosotis arvensis	N	B&I	●●●	
Myosotis azorica	N	HI		[28]

Myosotis scorpioides	N	B&I WNA	●●	[29]
Pentaglottis sempervirens	N	B&I	●●●	
Phacelia tanacetifolia	N	B&I	●●●	
Symphytum x uplandicum	N	B&I	●●●	
**Brassicaceae**				
Alliaria petiolata	N	B&I M&NE	●●	[35,125]
Alyssum sp.	A	EorWNA		[126]
Arabis caucasica	N	B&I		J. Owen
Armoracia rusticana	N	B&I WE	●●	[25]
Aubrieta deltoidea .	N	B&I		J. Owen
Barbarea stricta	N	WE		[25]
Barbarea verna	N	B&I ENA	●●	[32]
Barbarea vulgaris	N	ENA		[5]
Brassica napus	N	B&I WE	●●	[25,26]
Brassica nigra	?	ENA		[33]
Brassica oleracea	N,A	B&I ENA HI	●	[28,32,39]
Brassica rapa	N	B&I	●●●	
Calepina sp.	N	MED		[10,40]
Capsella bursa-pastoris	N	B&I WE MED ENA NZ	●●	[5,9,25,26,37]
Cardamine flexuosa	N	B&I		JRpercom
Cardamine pratensis	N	B&I WE	●	[72,115] Nickel
Descurainia sophia	N	WE		[25]
Diplotaxis tenuifolia	N	B&I	●●●	
Eruca vesicaria	N	B&I	●●●	

Erysimum cheiranthoides	N	WE WNA		[25,26,29]
Erysimum cheiri	N	B&I M&NE		J. Owen [35]
Hesperis matronalis	N	B&I WE	●●	[25]
Iberis sempervirens	A	B&I		J. Owen
Lepidium campestre	N	WE ENA		[25] [5]
Lepidium didymum	N	NZ		[37]
Lepidium draba	N	B&I, MED		[11] HMBL-England
Lepidium ruderale	N	WE		[25,26]
Lepidium virginicum	N	ENA		[5]
Lunaria annua	N	B&I	●●●	
Matthiola incana	N	WNA		[29]
Myagrum perfoliatum	N	MED		[8]
Nasturtium officinale	N	B&I	●●●	
Raphanus raphanistrum	N	B&I WNA HI	●●	[29,127]
Rapistrum sp.	N	MED		[40]
Rorippa amphibia	N	WE		[35]
Sinapis arvensis	N	B&I		HMBL-England
Sisymbrium altissimum	N	WE, ENA		[5,25]
Sisymbrium officinale	N	B&I	●●●	
Thlaspi arvense	N	F&S ENA		[33,36]
**Bromeliaceae**				
Fascicularia bicolor	N	B&I	●●●	
**Buxaceae**				

Buxus sempervirens	N	B&I	●●●	
Sarcococca confusa	N	B&I	●●●	
**Campanulaceae**				
Campanula patula	N	WE		[25]
Campanula persicifolia	N	WNA		[29]
Campanula glomerata	N	B&I M&NE	●●	[35]
Campanula latifolia	N	B&I	●●●	
Campanula medium	N	B&I ENA	●●	[5]
Campanula patula	N	F&S		[36]
Campanula persicifolia	N	F&S B&I	●●	[36]
Campanula portenschlagiana	N	B&I	●●●	
Campanula poscharskyana	N	B&I	●●●	
Campanula punctata	N	B&I	●●●	
Campanula pyramidalis	N	B&I	●●●	
Campanula rapunculus	N	B&I	●●●	
Campanula rotundifolia	N	F&S B&I	●●	[36]
Campanula trachelium	N	F&S B&I		[36] J. Owen
Jasione maritima	N	B&I MED	●●	[60]
Legousia sp.	N	MED		[8]
Lobelia cardinalis	N	B&I	●●●	
Lobelia gibbosa	N	B&I	●●●	
Phyteuma nigrum	N	MED		HMBL-Italy
**Cannabaceae**				

Cannabis sativa	N	MED, ENA		[128]
Humulus lupulus	N	WE		[25]
**Caprifoliaceae**				
Centranthus calcitrapae	N	MED		[60]
Centranthus ruber	N	B&I WNA	●●	[29]
Cephalaria gigantea	N	B&I	●●●	
Diervilla sp.	A	EorWNA		[126]
Dipsacus fullonum	N	B&I WE MED ENA	●	[5,25,63,98]
Knautia arvensis	N	F&S B&I WE	●	[26,36,63]
Knautia macedonica	N	B&I	●●●	
Knautia sylvatica	N	WE		[88]
Linnaea amabilis	N	B&I	●●●	
Linnaea x grandiflora	N	B&I	●●●	
Lomelosia sp.	A	MED		[7]
Lonicera caerulea	N	B&I	●●●	
Lonicera caprifolium	N	B&I	●●●	
Lonicera etrusca	N	B&I	●●●	
Lonicera japonica	N,A	B&I HI NZ	●●	[27,129]
Lonicera nitida	N	B&I	●●●	
Lonicera periclymenum	N	B&I	●●●	
Lonicera pileata	N	B&I	●●●	
Scabiosa atropurpurea	N	MED		[11]
Scabiosa columbaria	N	B&I	●●●	
Succisa pratensis	N	F&S B&I	●●	[36]

Succisella inflexa	N	B&I	●●●	
Symphoricarpos albus	N	B&I	●●●	
Valeriana excelsa	N	F&S		[36]
Valeriana officinalis	N	B&I WE	●●	[35]
Valeriana sambucifolia	N	F&S		[36]
Valerianella locusta	N	B&I	●●●	
Viburnum lantana	N	M&NE		[35]
Viburnum opulus	N	B&I	●●●	
Viburnum sargentii	N	B&I	●●●	
Viburnum tinus	N	B&I	●●●	
Viburnum x bodnantense	N	B&I	●●●	
Viburnum x carlcephalum	N	B&I	●●●	
Weigela sp.	N	WE ENA		[32,43]
**Caryophyllaceae**				
Agrostemma githago	N,A	B&I WE ENA	●●	[25,32]
Cerastium arvense	N	WE NZ		[25,37]
Cerastium brachypetalum	N	MED		[92]
Cerastium fontanum	N	B&I ENA	●●	[32]
Cerastium glomeratum	N	M&NE MED NZ		[6,35,37,40]
Cerastium holosteoides	N	B&I ENA NZ		[5,37]
Dianthus armeria	N	B&I	●●●	
Dianthus barbatus	N	B&I WNA	●●	[29]
Dianthus carthusianorum	N	B&I	●●●	
Dianthus caryophyllus	N	B&I WNA	●●	[29]

Dianthus chinensis	N	HI		[28]
Dianthus gratianopolitanus	N	B&I	●●●	
Dianthus plumarius	N	B&I	●●●	
Lychnis sp.	?	?		[57]
Moehringia trinervia	N	B&I WE	●●	[35]
Rabelera holostea	N	F&S B&I WE	●	[35–37]
Sagina apetala	N	WE		[35]
Saponaria officinalis	N	F&S B&I WE ENA WNA	●●	[29,32,35,36]
Scleranthus annuus	N	WE		[26]
Silene coronaria	N	B&I ENA	●●	[33]
Silene dioica	N	F&S B&I	●●	[36]
Silene flos-cuculi	N	F&S B&I WE	●	[36,72,115]
Silene gallica	N	MED		[92]
Silene latifolia	N	F&S B&I WE ENA	●●	[9,25,36,104]
Silene noctiflora	N	ENA		[5]
Silene uniflora	N	MED		[60]
Silene vulgaris	N	F&S B&I	●	[36,63]
Stellaria alsine	N	M&NE		[35]
Stellaria graminea	N,A	F&S B&I WE ENA	●	[36,63,87,88,130]
Stellaria media	N,A	F&S B&I MED ENA		[5,6,36,37,130]
Stellaria nemorum	N	WE		[25]
Stellaria palustris	N	F&S		[124]
**Celastraceae**				
Euonymus europaeus	N	B&I	●●●	

Euonymus fortunei	N	B&I	●●●	
Euonymus japonicus	N	B&I MED	●●	[75]
**Cercidiphyllaceae**				
Cercidiphyllum japonicum	N	B&I	●●●	
**Cistaceae**				
Cistus corbariensis	N	B&I	●●●	
Cistus creticus	N	MED		[69]
Cistus monspeliensis	N,A	MED		[106,131]
Cistus salvifolius	N	MED		[60]
Helianthemum nummularium	N	B&I WNA	●●	[29]
**Commelinaceae**				
Commelina diffusa	N	HI		[28]
Tradescantia fluminensi	N	WNA		[29]
**Convolvulaceae**				
Calystegia sepium	N	B&I WE	●●	[35]
Calystegia silvatica	N	NZ	●●	[37]
Convovulus acicularis	N	B&I MED		[63,97]
Convolvulus arvensis	N	B&I MED ENA WNA	●	[5,29,40,63]
Convolvulus cneorum	N	B&I	●●●	
Ipomea batatas	N	HI		[28]
Ipomoea indica	N	HI		[27]
Ipomoea muricata	N	WNA		[29]
**Coriariaceae**				

Coriaria sp.	A	NZ		[132]
**Cornaceae**				
Cornus alba	A	EorWNA		[126]
Cornus canadensis	N	ENA		[87]
Cornus racemosa	N	ENA		[58]
Cornus sanguinea	A	MED		[40]
Cornus suecia	N	F&S		[36]
**Crassulaceae**				
Crassula sarcocaulis	N	B&I	●●●	
Hylotelephium spectabile	N	B&I		J. Owen
Petrosedum rupestre	N	B&I		J. Owen
Phedimus spurius	N	B&I		J. Owen
Sedum acre	N	B&I	●●●	
Sedum album	N	B&I	●●●	
Umbilicus oppositifolius	N	B&I	●●●	
Umbilicus repestris	N	MED		[60]
**Cucurbitaceae**				
Cucumis sativus	N	B&I	●●●	
Cucurbita pepo	N	B&I	●●●	
**Cupressaceae**				
Cupressus sp.	A	MED		[74]
Hesperocyparis macrocarpa	A	WNA		VT-CA-2018-A
Juniperus communis	A	B&I		[133]

Juniperus oxycedrus	A	MED		[7]
Juniperus virginiana	A	ENA		VT-CT-2004-A
**Cyperaceae**				
Carex echinata	N	B&I	●●●	
Carex hirta	N	WE		[25]
Carex nigra	N	F&S		[134]
Carex panacea	N	B&I	●●●	
Kyllinga brevifolia	N	HI		[28]
Schoenoplectus tabernaemontani	N	F&S		[134]
Scirpus sp.	N	ENA		[58]
**Elaeagnaceae**				
Hippophae rhamnoides	N	B&I WE		[135] JRpercom
**Elaeocarpaceae**				
Crinodendron hookerianum	N	B&I	●●●	
**Equisetaceae**				
Equisetum arvense	N	F&S B&I MED ENA	●●	[58,86,121]
Equisetum silvaticum	N	F&S		[36]
**Ericaceae**				
Andromeda polifolia	N	B&I	●●●	
Arbusto sp.	A	MED		[40]
Arbutus unedo	N	B&I MED	●●	[69]
Calluna vulgaris	N,A	F&S B&I	●	[36,121,136,137]
Enkianthus sp.	A	EorWNA		[126]

Erica cinerea	N	B&I	●●●	
Erica scoparia	A	AZ		[138]
Erica tetralix	N	B&I	●●●	
Erica x darleyensis	N	B&I	●●●	
Leptecophylla tameiameiae	N	HI		[27]
Oxydendrum arboreum	N	B&I	●●●	
Pyrola minor	N	F&S		[36]
Vaccinium angustifolium	N,A	ENA		[87,139]
Vaccinium cylindraceum	N	AZ		[53]
Vaccinium macrocarpon	N,A	WNA		[140]
Vaccinium myrtillus	N	F&S B&I	●●	[36]
Vaccinium ovatum	N	B&I	●●●	
Vaccinium reticulatum	N	HI		[27]
Vaccinium uliginosum	N	F&S		[36]
Vaccinium vitis-idaea	N	F&S		[36]
**Escalloniaceae**				
Escallonia sp.	N	B&I		HMBL-England
**Euphorbiaceae**				
Euphorbia characias	N	B&I	●●●	
Euphorbia cyparissias	N	WE MED		[25,131]
Euphorbia dulcis	N	WE		[35]
Euphorbia griffithii	N	B&I	●●●	
Euphorbia purpurea	N	B&I	●●●	

Euphorbia serrata	N	WE		[83]
Euphorbia terracina	N	MED		[60]
Mercurialis perennis	N	B&I	●	[37]
Ricinus communis	N	B&I	●●●	
**Fabaceae**				
Acacia longifolia	N	MED		[60]
Anthyllis vulneraria	N	B&I	●●●	
Arachis hypogaea	A	MED		[85]
Argyrolobium biebersteinii	N	MED		[75]
Astragalus sp.	N	MED		[8]
Baptisia australis	N	B&I	●●●	
Bituminaria sp.	N	MED		[10]
Colutea sp.	A	EorWNA		[126]
Coronilla emerus	A	WE		[67]
Coronilla scorpioides	N	MED		[11]
Cytisus laniger	N	MED		[69]
Cytisus multiflora	N	MED		[60]
Cytisus praecox	N	B&I	●●●	
Cytisus scoparius	N,A	B&I MED WNA	●●	[60] VT-OR-2003-A
Cytisus × kewensis	N	B&I		J. Owen
Echinocystis fabacea	N	WNA		[29]
Galega officinalis	N	MED		[75]
Genista sp.	A	EorWNA		[126]
Glycine max	N	ENA		[141]

Hippocrepis sp.	N	MED		[10]
Lathyrus japonicus	N	ENA		VT-ME-2021-N
Lathyrus latifolius	A	WNA		VT-OR-2001-A
Lathyrus linifolius	N	B&I	●●●	
Lathyrus nissolia	N	B&I	●●●	
Lathyrus ochrus	N	MED		[11]
Lathyrus odoratus	N	ENA	●●	[5]
Lathyrus palustris	N	F&S		[134]
Lathyrus pratensis	N	F&S B&I	●	[36,37]
Lathyrus sativa	N	B&I	●●●	
Lathyrus silvestris	N	F&S		[36]
Lathyrus vernus	N	F&S		[36]
Lotus angustissimus	N	MED		[11]
Lotus corniculatus	N	F&S B&I WE MED ENA NZ	●	[9,36,37,60,88,142]
Lotus tetragonolobus	N	B&I		J. Owen
Lupinus arboreus	N	B&I	●●●	
Medicago cilaris	N	MED		[11]
Medicago littoralis	N	MED		[60]
Medicago lupulina	N	B&I WE		[26,63]
Medicago polymorpha	N	MED ENA HI		[5,6,28]
Medicago rigidula	N	MED		[11]
Medicago sativa	N,A	MED ENA WNA NZ		[5,29,70,143,144]
Medicago scutellata	N	MED		[11]
Melilotus alba Desv.	N	ENA		[145]

Melilotus indica	N	WNA		[29]
Melilotus officinalis	N	F&S MED ENA		[5,36,75]
Onobrychis viciifolia	N,A	WE EE MED		[74,83,146]
Ononis arvensis	N	F&S		[36]
Ononis repens	N	B&I	●●●	
Ononis spinosa	N	B&I	●●●	
Ornithopus compressus	N	MED		[92]
Phaseolus coccineus	N	B&I	●●●	
Phaseolus vulgaris	A	ENA		[141]
Pisum sativum	N,A	B&I ENA	●●	[32]
Psorolea bituminosa	N	MED		[75]
Robinia pseudoacacia	A	MED		[40]
Scorpiurus sp.	N	MED		[10]
Securigara sp.	N	MED		[10]
Senna corymbosa	N	B&I	●●●	
Trifolium alexandrinum	N	MED		[11]
Trifolium arvense	N	WE		[25]
Trifolium campestre	N	HI		[28]
Trifolium hybridum	N	F&S WE ENA		[5,26,36]
Trifolium medium	N	F&S ENA		[5,36]
Trifolium pratense	N,A	F&S B&I WE MED ENA NZ	●	[9,36,37,43,147]
Trifolium repens	N	F&S B&I MED ENA NZ	●	[5,6,36,63,148]
Trifolium rubens	N	B&I	●●●	
Trifolium spadiceum	N	F&S		[36]

Ulex europeaus	N,A	B&I	●	[149]
Vicia cracca	N	F&S B&I	●●	[36]
Vicia faba	N	MED		[9]
Vicia hirsuta	N	B&I WE	●	[37,82]
Vicia melanops	N	MED		[11]
Vicia sativa	N	B&I WE MED ENA WNA NZ	●	[25,32,37,60,110,131]
Vicia sepium	N	F&S B&I WE	●	[35–37]
Vicia tetrasperma	N	B&I	●●●	
Vicia villosa Roth	N,A	MED ENA WNA		[11,33] SEMC
**Fagaceae**				
Castanea sativa	N,A	B&I MED	●●	[44] [74]
Fagus sylvatica	N	B&I	●●●	
Quercus agrifolia	A	WNA		VT-CA-2018-A
Quercus cerris	A	MED		[150]
Quercus crenata	A	MED		[10]
Quercus ilex	A	MED		[7,10,22]
Quercus infectoria	A	MED		[150]
Quercus petraea.	A	MED		[40]
Quercus pubescens	A	MED		[45]
Quercus robur	N,A	B&I WE	●●	[67]
Quercus trojana	A	MED		[10]
**Gentianaceae**				
Centaurium erythraea	N	B&I WE HI	●	[28,63,82]

Gentiana asclepiadea	N	M&NE		[35]
Menyanthes trifoliata	N	F&S B&I	●●	[36]
**Geraniaceae**				
Erodium cicutarium	N	MED		[6,11,92]
Geranium carolinianum	N	HI		[28]
Geranium cinereum	N	B&I		J. Owen
Geranium dissectum	N,A	B&I MED	●●	[60]
Geranium macrorrhizum	N	B&I	●●●	
Geranium maculatum	N	B&I	●●●	
Geranium molle	N	MED	●●	[6,92]
Geranium platypetalum	N	B&I	●●●	
Geranium pratense	N	B&I	●●●	
Geranium pusillum	N	B&I WE	●●	[35]
Geranium pyrenaicum	N	B&I	●●●	
Geranium robertianum	N	B&I WE MED	●●	[35,60]
Geranium sanguineum	N	F&S, B&I		[124] HMBL-England
Geranium sylvaticum	N	F&S		[36]
Pelargonium graveolens	N	B&I WNA HI	●●	[27,29]
Pelargonium peltatum	N	WNA		[29]
Pelargonium × domesticum	N	WNA		[29]
Pelargonium × hortorum	N	WNA		[29]
**Grossulariaceae**				
Ribes nigrum	N	F&S B&I	●●	[36]

Ribes rubrum	N	B&I WNA	●●	[29]
Ribes sanguineum	N	B&I WE	●●	[35]
Ribes uva-crispa	N	B&I WE	●●	[35]
**Hydrangeaceae**				
Hydrangea macrophylla	N	WE		[25]
Hydrangea paniculata	N	B&I WNA	●●	[29]
Hydrangea petiolaris	N	B&I	●●●	
Philadelphus coronarius	N	B&I		J. Owen
**Hypericaceae**				
Hypericum androsaemum	N	B&I	●●●	
Hypericum calycinum	N	B&I	●●●	
Hypericum hirsutum	N	B&I		[63]
Hypericum maculatum	N	F&S		[36]
Hypericum moserianum	N	WNA HI		[28,29]
Hypericum olympicum	N	B&I	●●●	
Hypericum perforatum	N,A	F&S B&I WE MED	●	[7,26,36,37,63]
Hypericum pulchrum	N	B&I	●●●	
Hypericum tetrapterum	N	F&S, B&I	●●	[124]
**Iridaceae**				
Crocosmia × crocosmiiflora	N	B&I, HI		[28] HMBL-England
Gladiolus communis	N	B&I	●●●	
Hesperantha coccinea	N	B&I	●●●	
Iris germanica	N	MED		[75]

Iris pseudacorus	N	B&I	●●●	
Iris sibirica	N	B&I	●●●	
Iris versicolor	N	ENA		[87]
Libertia ixioides	N	B&I	●●●	
**Juglandaceae**				
Carya ovata	N	ENA		[5]
Juglans nigra	A	ENA		[151]
Juglans regia	?	MED		[152]
**Juncaceae**				
Juncus acutiflorus	N	B&I	●●●	
Juncus acutus	N	B&I	●●●	
Juncus effusus	N	B&I	●	[37]
Juncus inflexus	N	B&I	●●●	
Juncus gerardii	N	F&S		[134]
Juncus squarrosus	N	B&I	●●●	
**Juncaginaceae**				
Triglochin maritima	N	F&S		[36]
**Lamiaceae**				
Agastache foeniculum	N	B&I	●●●	
Agastache rugosa	N	B&I	●●●	
Ajuga reptans	N	B&I ENA	●	[5,63]
Callicarpa sp.	A	EorWNA		[126]
Dracocephalum parviflorum	N	ENA		[5]

Galeopsis bifida	N	F&S		[36]
Galeopsis speciosa	N	F&S		[36]
Galeopsis tetrahit	N	B&I WE		[35,37]
Glechoma hederacea	N	F&S B&I ENA	●●	[5,36]
Hyssopus officinalis ,	N	B&I WE	●	[115,153]
Lamium album	N	WE		[35]
Lamium amplexicaule	N	ENA		[5]
Lamium galeobdolon	N	B&I	●●●	
Lamium maculatum	N	WE MED		[60,82]
Lavandula angustifolia	N,A	B&I MED	●●	[154]
Lavandula stoechas	N,A	B&I MED	●●	[7,69]
Lavandula x intermedia	N	B&I		HMBL-England
Lycopus europaeus	N	F&S		[36]
Melissa officinalis	N	B&I	●●●	
Mentha aquatica	N	B&I M&NE	●●	[35]
Mentha arvensis	N	B&I M&NE	●●	[35]
Mentha spicata	N	B&I	●●●	
Mentha suaveolens	N	B&I	●●●	
Mentha x gracilis	N	B&I	●●●	
Mentha x piperita	N	B&I WE	●●	[155]
Mentha spicata	N	WNA		[29]
Monarda fistulosa	N	ENA		[5]
Nepeta cataria	N	B&I ENA	●●	[5]
Nepeta racemosa	N	B&I WNA		J. Owen [29]

Nepeta x faassenii	N	B&I	●●●	
Ocimum basilicum	N	B&I	●●●	
Origanum majorana	N	B&I MED	●●	[29]
Origanum vulgare	N	B&I WNA	●●●	VT-CA-2021-N
Physostegia virginiana	N	B&I	●●●	
Prunella vulgaris	N	F&S B&I WE		[35,36,63]
Salvia coccinea	N	WNA		[29]
Salvia elegans	N	B&I	●●●	
Salvia farinacea	N	B&I	●●●	
Salvia guaranitica	N	B&I	●●●	
Salvia longispicata	N	B&I	●●●	
Salvia mircophylla	N	B&I	●●●	
Salvia nemorosa	N	B&I	●●●	
Salvia officialis	N	B&I WNA	●●	[29]
Salvia rosmarinus	N	B&I MED WNA	●	[8,29,120]
Salvia splendens	N	WE		[25]
Salvia x jamensis	N	B&I	●●●	
Salvia yangii	N	B&I	●●●	
Satureja hortensis	N	B&I	●●●	
Satureja montana	N	B&I	●●●	
Scutellaria galericulata	N	F&S		[36]
Stachys ajugoides	N	WNA		[29]
Stachys bullata	N	WNA		[29]
Stachys byzantina	N	B&I	●●●	JRpercom

Stachys germanica	N	B&I		[156]
Stachys palustris	N	M&NE		[35]
Stachys rigida	N	WNA		RKpercom
Stachys sylvatica	N	B&I WE		[35,37]
Teucrium scorodonia	N	B&I	●●●	
Thymus citriodorus	N	B&I	●●●	
Thymus mastichina	A	MED		[7]
Thymus serpyllum	N	B&I	●●●	
Thymus vulgaris	N	F&S WNA	●●	[29]
**Lauraceae**				
Laurus nobilis	N	B&I	●●●	
**Liliaceae**				
Lilium martagon	N	B&I	●●●	
Tulipa sp.	N	B&I		J. Owen
**Linaceae**				
Linum catharticum	N	B&I	●●●	
Linum usitatissimum	N,A	F&S B&I	●●	[157]
**Lythraceae**				
Lythrum maritimum	N	ENA HI		[28,32]
Lythrum salicaria	N	F&S B&I M&NE ENA	●●	[35,36,158]
**Magnoliaceae**				
Magnolia denudata	N	B&I	●●●	
Magnolia liliiflora	N	B&I	●●●	

Magnolia stellata	N	B&I	●●●	
**Malvaceae**				
Alcea rosea	N	B&I, WNA		[29] J. Owen
Althaea cannabina	N	B&I	●●●	
Althaea officinalis	?	WE		[72]
Gossypium hirsutum	?	EE		[146]
Hibiscus syriacus	N	B&I	●●●	
Hibiscus tiliaceus	N	HI		[28]
Kokia drynarioides	N	HI		[27]
Malva moschata	N	B&I	●	[63]
Malva neglecta	N	WE		[25]
Malva parviflora	N	WNA		[29]
Malva sylvestris	N	B&I MED	●●	[11]
Modiola caroliniana	N	HI		[28]
Tilia platyphyllos	N	F&S		[121]
**Melastomataceae**				
Tibouchina semidecandra	N	HI		[28]
**Montiaceae**				
Claytonia virginica	N	ENA		[5]
**Moraceae**				
Ficus carica	N,A	B&I WE	●●	[36,67,72]
Morus alba	A	WE		[67]
**Musaceae**				

Musa sp.	A	MED		[85]
**Myricaceae**				
Comptonia peregrina	A	ENA		VT-WI-2000-A
Myrica faya	A	AZ		[138]
Myrica gale	N	F&S B&I	●●	[36,137]
**Myrtaceae**				
Calothamnus graniticus	N	B&I	●●●	
Eucalyptus amygdalina	N	B&I	●●●	
Leptospermum laevigatum	N	WNA		[29]
Leptospermum scoparium	N	B&I	●●●	
Metrosideros collina	N	HI		[28]
Myrtus communis	N,A	B&I MED	●●	[68]
**Nartheciaceae**				
Narthecium ossifragum	N	B&I		HMBL-Scotland
**Nymphaeaceae**				
Nymphaea sp.	N	B&I		[39]
**Oleaceae**				
Forsythia suspensa	N	B&I	●●●	
Forsythia × intermedia	N	B&I		J. Owen
Fraxinus excelsior	N	F&S B&I	●●	[121]
Fraxinus ornus	A	MED		[10]
Fraxinus pennsylvanica	N	ENA		[5]
Jasminum humile	N	B&I	●●●	

Jasminum nudiflorum	N	B&I	●●●	
Jasminum officinale	N	B&I	●●●	
Ligustrum ovalifolium	N	B&I ENA	●●	[5]
Ligustrum vulgare	N	B&I ENA	●●	[5]
Olea europaea	N,A	B&I MED	●●	[6,7,78,110,159]
Osmanthus delavayi	N	B&I	●●●	
Syringa vulgaris	N	F&S B&I ENA	●●	[5,36]
**Onagraceae**				
Chamaenerion angustifolium	N,A	F&S B&I WE ENA WNA	●	[36,87,91,160] VT-WA-2003-N
Circaea canadensis	N	B&I	●●●	
Epilobium alsinifolium	N	B&I	●●●	
Epilobium ciliatum	N	B&I	●●●	
Epilobium hirsutum	N	B&I	●●●	
Epilobium lanceolatum	N	B&I	●●●	
Epilobium montanum	N	F&S B&I WE	●	[35–37]
Epilobium obscurum	N	B&I M&NE	●●	[35]
Epilobium parviflorum	N	B&I M&NE	●●	[35]
Epilobium roseum	N	M&NE		[35]
Epilobium tetragonum	N	B&I	●●●	
Fuchsia arborescens	N	HI		[27]
Fuchsia magellanica	N	B&I HI	●●	[28]
Fuchsia microphylla	N	B&I	●●●	
Fuchsia procumbens	N	B&I	●●●	
Fuchsia triphylla	N	WNA		[29]

Oenothera biennis	N,A	B&I, WE ENA	●●	[26,161] HMBL-England
Oenothera glaziovana	N	NZ		[37]
Oenothera lindheimeri	N	B&I	●●●	
Oenothera odorata	N	HI		[42]
Oenothera stricta	N	HI		[28]
**Orchidaceae**				
Dactylorhiza fuchsii	N	B&I	●●●	
Dactylorhiza maculata	N	B&I	●●●	
Neottia ovata	N	B&I		[37]
**Orobanchaceae**				
Bellardia sp.	N	MED		[8]
Euphrasia officinalis	N	B&I		CH-England-2019-N
Melampyrum nemorosum	N	F&S		[36]
Melampyrum pratense	N	F&S, MED		[36] HMBL-Italy
Melampyrum sylvaticum	N	F&S		[36]
Odontites vernus	N	B&I		CH-England-2019-N
Pedicularis palustris	N	WE		[88]
Rhinanthus minor	N	F&S B&I WE ENA	●●	[35,36,87]
Rhinanthus serotinus	N	F&S		[36]
**Osmundaceae**				
Osmunda regalis	N	B&I	●●●	
**Oxalidaceae**				
Oxalis acetosella	N	WE		[88]

Oxalis corniculata	N	HI		[28]
Oxalis stricta	N	ENA		[104]
**Papaveraceae**				
Chelidonium majus	N	WE		[25]
Eschscholzia californica	N	B&I WNA	●●	[29]
Fumaria sp.	N	MED		[10]
Papaver cambricum	N	B&I	●●●	
Papaver dubium	N	WE		[26]
Papaver nudicaule	N	B&I	●●●	
Papaver orientale	N	B&I ENA WNA	●●	[5,29]
Papaver rhoeas	N	B&I WNA	●●	[29]
Papaver somniferum	N	B&I WE	●●	[25]
Roemeria argemone	N	WE		[26]
Stylophorum diphyllum	N	B&I	●●●	
**Phrymaceae**				
Diplacus aurantiacus	N	B&I	●●●	
Erythranthe guttata	N	WNA		[162]
Erythranthe lutea	N	B&I	●●●	
**Phyllanthaceae**				
Phyllanthus niruri	N	B&I	●●●	
**Pinaceae**				
Abies balsamea	N	ENA		[87]
Picea glauca	N	ENA		[87]

Pinus banksiana	A	ENA		VT-MI-2001-A
Pinus contorta	N,A	B&I WNA		[137] VT-CA-2018-A
Pinus halepensis	A	MED		[45]
Pinus ponderosa	N,A	WNA		[163] VT-OR-2003-A
Pinus radiata	A	WNA		VT-CA-2018-A
Pinus resinosa	A	ENA		VT-MI-2001-A
Pinus strobus	N,A	ENA		[33] VT-NH-2020-N
Pinus sylvestris	A	ENA		[164]
Pinus virginiana	A	ENA		VT-MA-2006-A
**Pittosporaceae**				
Billardiera heterophylla	N	B&I	●●●	
Pittosporum tenuifolium	N	B&I	●●●	
Pittosporum undulatum	A	AZ		[138]
**Plantaginaceae**				
Antirrhinum majus	N	B&I		J. Owen
Hebe rakaiensis	N	B&I	●●●	
Hebe salicifolia	N	B&I HI	●●	[28]
Linaria vulgaris	N	F&S, WE		[35,124]
Plantago coronopus	N	MED		[69]
Plantago lagopus	N	WE MED		[11,25]
Plantago lanceolata	N,A	B&I WE MED ENA WNA HI NZ	●	[25,28,29,32,37,40,60,63]
Plantago major	N,A	F&S B&I WE ENA NZ	●●	[5,25,36,37]
Plantago media	N	B&I WE	●●	[25]

Plantago maritima	N	F&S B&I	●●	[36]
Plantago rugelii	?	ENA		[33]
Veronica agrestis	N	WE		[35]
Veronica arvensis	N	B&I WE MED	●●	[25,40]
Veronica beccabunga	N	M&NE		[35]
Veronica chamaedrys	N	F&S B&I WE	●	[35,36,63]
Veronica hederifolia	N	B&I		J. Owen
Veronica longifolia	N	M&NE		[35]
Veronica officinalis	N	WE		[35]
Veronica peduncularis	N	B&I	●●●	
Veronica pinguifolia	N	B&I		HMBL-England
Veronica peregrina	N	ENA		[5]
Veronica persica	N	B&I		[63]
Veronica plebeia	N	HI		[28]
Veronica serpyllifolia	N	B&I	●●●	
Veronica spicata	N	B&I		J. Owen
Veronica urticifolia	N	MED		HMBL-Italy
Veronicastrum sp.	N	B&I		HMBL-England
**Platanaceae**				
Platanus occidentalis	N	ENA		[5]
**Plumbaginaceae**				
Armeria maritima	N	B&I	●●●	
**Poaceae**				

Agrostis canina	N	B&I		[37]
Agrostis capillaris	N	B&I, WE MED	●	[26,37,75]
Agrostis gigantea	N,A	B&I ENA		[32,37]
Agrostis stolonifera	N	B&I ENA NZ	●	[5,37]
Alopecurus myosuroides	N	B&I	●●●	
Alopercurus pratensis	N	F&S B&I NZ	●●	[36,37]
Anemanthele lessoniana	N	B&I	●●●	
Anisantha sp.	N	MED		[10]
Anthoxanthum odoratum	N	B&I ENA NZ	●●	[33,37]
Apera spica-venti	N	WE		[26]
Arrhenatherum elatius	N,A	B&I MED ENA NZ	●●	[5,37,165]
Arundo donax	N	MED		[9]
Avena barbata	N	MED		[75]
Avena fatua	N	WNA		[110]
Avena sativa	N,A	F&S ENA		[5,36,157]
Avena sterilis	N	MED		[6,11]
Brachiaria mutica	N	HI		[28]
Brachypodium pinnatum	N	WE		[166]
Bromus hordeaceus	N	B&I WE MED NZ		[11,25,37,75]
Bromus secalinus	N	ENA		[104]
Bromus sterilis	N	MED		[6,11]
Bromus tectorum	N	WE		[26]
Bromus willenowii	N	NZ		[37]
Calamagrostis sp.	N	F&S		[36]

Coix lachryma-jobi	N	HI		[28]
Corynephorus canescens	N	WE		[26]
Cyinosurus sp.	?	MED		[97]
Cynodon dactylon	N	MED HI		[28,92]
Cynosurus cristatus	N	B&I NZ	●●	[37]
Dactylis glomerata	N	B&I MED ENA WNA HI NZ	●	[5,6,28,37,60]
Dasypyrum villosum	N	MED		[11]
Deschampsia cespitosa	N	B&I	●●●	
Dichanthelium dichotomum	N	ENA		VT-NH-2020-N
Digitaria horizontalis	N	HI		[28]
Elymus repens	N	B&I WE ENA NZ	●●	[5,25,37]
Festuca arundinacea	N	F&S B&I NZ	●●	[37,134]
Festuca glauca	N	B&I WE	●●	[26]
Festuca ovina	N	B&I	●●●	[26]
Festuca rubra	N	B&I WE	●●	[26]
Helictotrichon pratense	N	B&I	●●●	
Holcus lanatus	N,A	B&I WE HI NZ	●	[25,28,37,167]
Holcus mollis	N	B&I WE NZ	●●	[37,72]
Hordeum brachyantherum	N	WNA		[110]
Hordeum bulbosum	N	MED		[75]
Hordeum murinum	N	MED WNA NZ		[6,29,37,75]
Hordeum vulgare	N,A	F&S ENA		[5,32,36,157]
Lagurus ovatus	N	MED		[60]
Leymus arenarius	N	B&I	●●●	

Lolium multiflorum	N,A	ENA		[32]
Lolium perenne	N	B&I WE MED NZ	●	[11,26,37]
Melica uniflora	N	B&I	●●●	
Milium effusum	N	WE		[88]
Molinia caerulea	N	B&I	●●●	
Muhlenbergia rigens	N	B&I	●●●	
Nassella tenuissima	N	B&I	●●●	
Panicum virgatum	N	B&I	●●●	
Pennisetum clandestinum	N	B&I	●●●	
Phalaris aquatica	N	NZ		[37]
Phalaris arundinacea	N	B&I	●●●	
Phleum pratense	N	F&S B&I ENA NZ	●●	[5,36,37]
Phragmites australis	N	F&S B&I WE MED	●●	[88,134,168]
Poa annua	N	B&I MED	●●	[6]
Poa pratensis	N	B&I WE ENA NZ	●	[5,26,37]
Poa trivialis	N	B&I NZ	●	[37]
Rostraria sp.	?	MED		[97]
Sacciolepis indica	N	HI		[28]
Secale cereale	N	F&S WE		[25,36]
Sorghum halepense	N	Italy		[98]
x Triticosecale	A	EE		[169]
Triticum aestivum	N,A	F&S MED EorWNA		[32,36,65,98]
Triticum monococcum	N	EE		[170]
Triticum polonicum	N	EE		[170]

Zea mays	N,A	ENA		[5,32]
**Polemoniaceae**				
Phlox douglasii	N	B&I	●●●	
Phlox paniculata	N	B&I	●●●	
Phlox subulata	N	B&I	●●●	
Polemonium caeruleum	N	F&S, B&I	●●	[124]
**Polygonaceae**				
Bistorta officinalis	N	B&I WE	●●	[35]
Bistorta vivipara	N	F&S		[36]
Fagopyrum esculentum	A	ENA		[58]
Fallopia baldschuanica	N	B&I	●●●	
Fallopia convovulus	N	WE		[35]
Persicaria amphibia	N	F&S, M&NE		[35,124]
Persicaria lapathifolia	N	B&I	●●●	
Persecaria maculosa	N	B&I	●	[37]
Persicaria odorata	N	B&I	●●●	
Polygonum aviculare	N	F&S WE MED		[9,35,36]
Polygonum lapathifolium	N	F&S		[36]
Reynoutria multiflora	N	B&I	●●●	
Rheum rhaponticum	N	B&I		J. Owen
Rheum x hybridum (?)	N	B&I	●●	[28,171]
Rumex acetosa	N	F&S B&I WE MED	●	[25,26,36,60,63]
Rumex acetosella	N	F&S B&I WE ENA WNA HI	●●	[26,28,36,87,172]

Rumex aquaticus	N	F&S		[36]
Rumex bucephalophorus	N	MED		[6,92]
Rumex conglomeratus	N	B&I WE WNA	●●	[29,35]
Rumex crispis	N	F&S, B&I WE MED ENA WNA	●●	[9,29,87,102,124]
Rumex hydrolapathum	N	WE		[35]
Rumex induratus	N	MED		[92]
Rumex longifolius .	N	F&S		[36]
Rumex occidentalis	?	ENA		[33]
Rumex obtusifolius	N,A	B&I WE ENA	●	[5,25,173]
Rumex sanguineus	N	B&I	●●●	
**Polypodiaceae**				
Dryopteris carthusiana	N	ENA		[87]
Ecballium elaterium	N	MED		[8]
Pteridium aquilinum	N,A	F&S B&I EE NZ	●	[36,174–176]
**Primulaceae**				
Anagallis arvensis	N	MED HI		[11,28]
Glaux maritima	N	F&S		[36]
Lysimachia arvensis	N	Italy		[11]
Lysimachia ciliata	N	B&I		HMBL-England
Lysimachia clethroides	N	B&I	●●●	
Lysimachia europaea	N	F&S		[36]
Lysimachia foemina	N	MED		[6]
Lysimachia nemorum	N	B&I		[37]

Lysimachia nummularia	N	B&I, WE		[35] J. Owen
Lysimachia punctata	N	B&I	●●●	
Lysimachia thyrsiflora	N	F&S		[36]
Lysimachia vulgaris	N	F&S B&I WE	●●	[36,72]
Primula auricula	N	B&I	●●●	
Primula denticulata	N	B&I	●●●	
Primula prolifera	N	B&I	●●●	
Primula rosea	N	WE		[35]
Primula veris	N	B&I	●	[15,63]
Primula vialii	N	B&I	●●●	
Primula vulgaris	N	B&I	●	[37]
**Proteaceae**				
Adenanthos cuneatus	N	B&I	●●●	
Grevillea rosmarinifolia	N	B&I	●●●	
Lambertia rariflora	N	B&I	●●●	
**Ranunculaceae**				
Adonis sp.	N	MED		[8]
Anemonoides sylvestris	N	M&N Europe		[35]
Aquilegia canadensis	N	ENA		[5]
Aquilegia chrysantha	N	WNA		[29]
Aquilegia vulgaris	N	B&I	●●●	
Caltha palustris	N	F&S B&I WE	●	[35,36,63]
Clematis cirrhosa	N	B&I	●●●	

Clematis montana	N	B&I	●●●	
Clematis tangutica	N	B&I	●●●	
Clematis vitalba	N	B&I WE MED	●●	[75,83]
Eriocapitella hupehensis	N	B&I	●●●	
Nigella damascena	N	B&I	●●●	
Ranunculus abortivus	N	ENA		[5]
Ranunculus acris	N	F&S B&I WE ENA	●●	[25,36,83,87]
Ranunculus arvensis	N	WE		[88]
Ranunculus auricomus	N	B&I	●●●	
Ranunculus bulbosus	N	B&I WE	●●	[26]
Ranunculus cymbalaria	N	B&I	●●●	
Ranunculus flammula	N	F&S B&I	●●	[36]
Ranunculus lingua	N	B&I M&NE	●●	[35]
Ranunculus repens	N,A	F&S B&I WE MED WNA	●	[29,35,36,60,63]
Ranunculus sceleratus	N	B&I	●●●	
Thalictrum flavum	N	M&NE		[35]
Trollius chinensis	N	B&I	●●●	
**Resedaceae**				
Reseda lutea	N	WE		[25]
**Rhamnaceae**				
Ceanothus thyrsiflorus	N	B&I		HMBL-England
Rhamnus alaternus	N,A	B&I MED	●●	[10]
Rhamnus cathartica	A	ENA		[177]

Rosaceae

Agrimonia eupatoria	N	B&I ENA	●	[58,63]
Alchemilla glabra	N	B&I		[63]
Alchemilla mollis	N	B&I	●●●	
Alchemilla vulgaris	N	F&S		[36]
Amelanchier spicata	N	F&S		[36]
Argentina anserina	N	B&I	●●●	
Aronia sp.	?	ENA		[178]
Aruncus sylvester	N	B&I		J. Owen
Chaenomeles japonica	N	WNA		[29]
Chaenomeles speciosa	N	B&I	●●●	
Comarum palustre	N	B&I	●●●	
Cotoneaster conspicuus	N	B&I	●●●	
Cotoneaster franchetii	N	B&I	●●●	
Cotoneaster horizontalis	N	B&I	●●●	
Cotoneaster salicifolius	N	B&I	●●●	
Crataegus monogyna	N,A	B&I M&NE	●	[35,179]
Cydonia oblonga	N	B&I	●●●	
Dasiphora fruticosa	N	B&I	●●●	
Filipendula ulmaria	N,A	F&S B&I WE	●	[35–37,72]
Filipendula vulgaris	N	B&I		HMBL-England
Fragaria chiloensis	N	WNA HI		[27,29]
Fragaria moschata	N	WE		[35]
Fragaria vesca	N	F&S, B&I	●●	[36]

Fragaria virginiana	N	ENA		[180]
Fragaria x ananassa	N,A	F&S B&I WE ENA HI Reunion		[5,36,42,57]
Geum aleppicum	N	ENA		[181]
Geum coccineum	N	B&I	●●●	
Geum quellyon	N	B&I	●●●	
Geum rivale	N	F&S B&I	●	[36,63]
Geum ubanum	N	F&S B&I WE MED	●	[9,25,36,37]
Kerria japonica .	A	EorWNA		[126]
Malus domestica	N,A	B&I WE ENA	●●	[32,58,182]
Malus sylvestris	N,A	B&I ENA	●	[183,184]
Margyricarpus pinnatus	N	B&I	●●●	
Photinia x fraseri	N	B&I	●●●	
Physocarpus opulifolius	N	B&I	●●●	
Potentilla anserina	N	F&S WE		[35,36]
Potentilla argentea	N	F&S		[36]
Potentilla canadensis	N	ENA		[5]
Potentilla erecta	N	F&S B&I WE	●●	[25,36,137]
Potentilla norvegica	N	F&S, ENA		[104,124]
Potentilla palustris	N	F&S		[36]
Potentilla recta	A	ENA		[185]
Potentilla reptans	N	B&I		[63]
Potentilla sterilis	N	B&I	●	[37]
Prunus americana	?	ENA		[31]
Prunus armeniaca	N,A	ENA WNA		[29,186]

Prunus avium	N	B&I ENA	●●	[58]
Prunus cerasifera	N	B&I		J. Owen
Prunus cerasus	N,A	M&NE ENA		[35,187]
Prunus domestica	N,A	B&I WE MED ENA	●●	[25,74,186]
Prunus dulcis	N,A	B&I MED	●●	[7,78]
Prunus laurocerasus	A	WE		[188]
Prunus padus	N	F&S		[36]
Prunus pensylvanica	?	ENA		[189]
Prunus persica	A	EE ENA		[58,182]
Prunus serrulata	N	B&I		J. Owen
Prunus spinosa	N	B&I	●●●	
Prunus virginiana	A	ENA		[41] VT-NH-2006-A
Pyracantha rogersiana	N	B&I	●●●	
Pyrus communis	N,A	WE MED ENA		[58,74,82]
Pyrus spinosa	A	MED		[45]
Rosa arvensis	N	B&I	●●●	
Rosa canina	N	F&S B&I WE	●●	[25,36]
Rosa chinensis	N	ENA		[32]
Rosa foetida	N	B&I	●●●	
Rosa gallica	N	B&I	●●●	
Rosa hybrids (domesticated)	N	F&S WE ENA		[5,36,82]
Rosa multiflora	N	ENA	●●	[126]
Rosa nitida	N	ENA		[87]
Rosa pimpinellifolia	N	B&I	●●●	

Rosa rubiginosa	N	B&I		HMBL-England
Rosa rugosa	N	B&I ENA	●●	[87]
Rosa setigera	N	B&I	●●●	
Rosa × damascena	A	EE		[190]
Rubus allegheniensis	N	ENA		[5]
Rubus arcticus	N	F&S		[36]
Rubus argutus .	N	HI		[28]
Rubus armeniacus	N	B&I		[191]
Rubus caesius	N	B&I M&NE	●●	[35]
Rubus canadensis	N,A	ENA		[5,32]
Rubus canescens .	?	MED		[97]
Rubus chamaemorus	N	F&S B&I	●●	[36]
Rubus fruticosus	N	B&I NZ	●	[37]
Rubus hispidus	N	ENA		[87]
Rubus idaeus	N	F&S B&I ENA	●●	[36,87]
Rubus laciniatus	N	B&I		J. Owen
Rubus occidentalis	N,A	ENA		[32]
Rubus parviflorus	N	WNA		[29]
Rubus procerus	N	WNA		[29]
Rubus rosaceus	?	ENA		[33]
Rubus saxatilis	N	F&S		[36]
Rubus ulmifolius	N	B&I	●●●	
Rubus ursinus	N	WNA		VT-CA-2021-N
Rubus vitifolius	N	WNA		[29]

Rubus × loganobaccus	N	B&I	●	[191]
Sanguisorba minor	N	B&I MED	●●	[97]
Sanguisorba officinalis	N	B&I	●●●	
Sanguisorba verrucosa	N	MED		[92]
Sorbaria sorbifolia	N	B&I	●●●	
Sorbus americana	N	ENA		[87]
Sorbus aucuparia	N	F&S B&I	●●	[36]
Spiraea alba	N	ENA		[102]
Spiraea arguta	N	B&I	●●●	
Spiraea cantoniensis	N	WE		[43]
Spiraea douglasii	N	B&I	●●●	
Spiraea japonica	N	B&I	●●●	
Spiraea nipponica	N	B&I	●●●	
Spiraea splendens	N	WNA		VT-WA-2003-N
Spiraea vanhouttei	N	B&I		J. Owen
**Rubiaceae**				
Coprosma ernodeoides	N	HI		[28]
Coprosma rhynchocarpa	N	HI		[28]
Crucianella maritima	N	MED		[60]
Cruciata laevipes	N	B&I	●●●	
Galium aparine	N	B&I WE MED ENA WNA	●	[9,29,35,37,192]
Galium asprellum	N	ENA		[104]
Galium boreale	N	F&S		[36]
Galium mollugo	N	F&S B&I WE MED ENA	●●	[9,36,88] VT-NH-2020-N

Galium palustre	N	F&S B&I	●●	[36]
Galium saxatile	N	B&I	●●●	
Galium sylvaticum	N	WE ENA		[5,35]
Galium uliginosum	N	F&S		[36]
Galium verum	N	F&S B&I WE ENA	●●	[5,35,36]
Palicourea elata	N	B&I	●●●	
Rubia peregrina	N	B&I	●●●	
Rubia tinctorum	N	B&I		JRpercom
Sherardia arvensis	N	MED		[10,78]
Theligonum sp.	N	MED		[10]
**Rutaceae**				
Citrus sp.	A	MED		[74]
Phellodendron sp.	A	EorWNA		[126]
Ruta sp.	N	MED		[10]
**Salicaceae**				
Populus nigra	N	MED		[75]
Populus tremula	N	F&S		[36]
Salix alba	N,A	WE MED EorWNA		[26,67,75,126]
Salix babylonica	N,A	B&I MED	●●	[75,123]
Salix caprea	N,A	F&S B&I WE	●●	[67,121]
Salix cinerea	N	F&S		[121]
Salix fragilis	N	WE		[26]
Salix phylicifolia	N	F&S		[36]

Salix purpurea	A	WE		[67]
Salix repens	N	F&S		[121]
Salix viminalis	N	F&S EE		[121,193]
**Santalaceae**				
Thesium sp.	N	MED		[40]
**Sapindaceae**				
Acer campestre	N,A	B&I MED	●●	[123]
Acer negundo	N	ENA		VT-WI-1995-A
Acer palmatum	N	B&I	●●●	
Acer platanoides	N	B&I	●●●	
Acer pseudoplatanus	N	B&I	●●●	
Acer pycnanthum	N	B&I	●●●	
Acer rubrum	N	ENA		VT-NH-2020-N
Acer spicatum	N	ENA		[87]
Aesculus hippocastanum	N	B&I	●●●	
Aesculus parviflora	A	EorWNA		[126]
Hippuris vulgaris	N	B&I	●●●	
**Sapotaceae**				
Sideroxylon sp.	N	HI		[27]
**Saururaceae**				
Houttuynia cordata	N	B&I		HMBL-England
**Saxifragaceae**				
Bergenia crassifolia	N	B&I		J. Owen

Chrysosplenium oppositifolium	N	B&I	●●●	
Heuchera sanguinea	N	WNA		[29]
Tellima grandiflora	N	B&I	●●●	
**Scrophulariaceae**				
Buddleja alternifolia	N	B&I	●●●	
Buddleia davidii	N	B&I	●	[194]
Buddleja globosa	N	B&I	●●●	
Linaria purpurea	N	B&I	●●●	
Phygelius capensis	N	B&I	●●●	
Scrophularia auriculata	N	B&I	●●●	
Scrophularia californica	N	WNA		[29]
Verbascum phoeniceum	N	B&I		HMBL-England
Verbascum thapsus	?	ENA		[33]
Scrophularia nodosa	N	WE		[35]
**Solanaceae**				
Capsicum annuum	N	B&I WE	●●	[83]
Capsicum chinense	N	B&I	●●●	
Cestrum elegans	N	WNA		[29]
Lycoperscion esculentum	N	EE EorWNA HI		[27,56,65]
Nicotiana tabacum	?	EorWNA		[65]
Physalis alkekengi	N	ENA		[104]
Physalis peruviana	N	HI		[28]
Solanum dulcamara	N,A	WE ENA		[87,195] AMNH

Solanum lycopersicum	N	B&I	●●●	
Solanum nigrum	N	WE		[195]
Solanum tuberosum	N,A	B&I EE MED Uzbekistan ENA	●●	[56,62,196,197]
**Staphyleaceae**				
Staphylea sp.	A	EorWNA		[126]
**Tamaricaceae**				
Tamarix tetrandra	N	B&I	●●●	
**Taxaceae**				
Taxus baccata	N	B&I	●●●	
**Thymelaeaceae**				
Daphne mezereum	N	B&I		J. Owen
Dirca palustris	A	EorWNA		[126]
Wikstroemia phillyreifolia	N	HI		[28]
**Tropaeolaceae**				
Tropaeolum majus	N	B&I	●●●	
**Ulmaceae**				
Celtis occidentalis	A	ENA		VT-IL-2001-A
Ulmus glabra	N	F&S		[121]
Ulmus laevis	A	WE		[67]
Ulmus minor	A	WE MED		[45,67]
**Urticaceae**				
Parietaria judaica	A	MED		[64]
Parietaria officinalis	N	WE		[82]

Pipturus sp.	N	HI		[27]
Urtica dioica	N,A	F&S B&I WE ENA	●	[36,63,72,87]
Urtica urens	N	WE		[35]
**Verbenaceae**				
Aloysia citrodora	N	B&I	●●●	
Stachytarpheta sp.	N	HI		[127]
Verbena bonariensis	N	B&I	●●●	
Verbena hastata	N	B&I	●●●	
Verbena litoralis	N	HI		[28]
Verbena rigida	N	WNA		[29]
**Violaceae**				
Viola canina	N	F&S B&I	●●	[36]
Viola epipsila	N	F&S		[36]
Viola odorata	N	WNA		[29]
Viola palustris	N	M&NE		[35]
Viola riviniana	N	B&I	●	[37]
Viola tricolor	N	WE, ENA		[5,35]
Viola x wittrockiana	N	B&I	●●●	
**Vitaceae**				
Parthenocissus henryana	N	B&I	●●●	
Parthenocissus quinquefolia	N	B&I	●●●	
Vitis vinifera	N,A	WE EE MED		[40,78,198–200]
**Zingiberaceae**				

Hedychium coronarium	N	HI		[27]

Footnotes for Table 1

Stage: N = nymph, A = adult, N,A = nymph and adult, ? = unknown

Geographic area: F&S = Finland and Scandinavia, B&I = Britain and Ireland, WE = Western Europe, EE = Eastern Europe, MED = Mediterranean Basin, ENA = Eastern North America, WNA = Western North America, HI = Hawaii, NZ = New Zealand, AZ = Azores Islands, ? = unknown. Western Europe includes France, Belgium, the Netherlands, Luxembourg, Germany, Switzerland, the Czech Republic, Austria and Slovenia. Eastern Europe includes the Baltic countries, Poland, Slovakia, Hungary, Serbia, Kosovo, North Macedonia, Romania, Bulgaria, Belarus, Ukraine, Moldova, Russia and Georgia. The Mediterranean Basin includes all countries bordering the Mediterranean except France and Slovenia (but, as a partial exception, includes Corsica). Eastern North America includes the USA and Canada east of the 100th meridian. Western North America includes the USA and Canada west of the 110th meridian. A number of Noury's [35] listings are from a source covering "Middle and Northern Europe", a category that does not fit our arbitrary divisions. These records are recorded as M&NE. A few North American references do not specify which section of the continent. These are recorded as EorWNA.

Citizen science observations: ●●● = new to the scientific record, ●● = new to B&I, ● = confirmation of earlier B&I record(s). Note that because they are host records new to science, there are no additional source citations for ●●● host species.

Sources: Published sources are cited by numbered references. Unpublished sources are cited as follows. Thirty unpublished observations by VT are referenced in the following format: VT-XX-YYYY-Z, where XX represents the standard two letter postal abbreviations for the American states, YYYY

represents the year of observation, and Z represents the stage observed (N or A). Three unpublished observations by CH are referenced in an analogous format substituting “England” for the XX state abbreviation: CH-England-2019-N. Twenty-five unpublished nymphal observations provided by Saskia Hogenhout, Sam Mugford, Roberto Biello and Qun Liu (John Innes Centre) dating from 2019-2021 are referenced as HMBL-XX, where XX is England, Scotland or Italy. Thirty-three English nymphal records underlying the work of Jennifer Owen [38] and recovered from her archived papers are referenced simply as J. Owen. Five adult records are based on pin label information from the following collections: American Museum of Natural History (AMNH), University of California Riverside Entomology Research Museum (UCRC) and Snow Entomological Museum Collection (SEMC), listed by their respective abbreviations. Three nymphal observations from Scotland by John Raven (University of Dundee) and three from California by Richard Karban (University of California, Davis), all from 2022, are referenced as personal communications: JRpercom and RKpercom.

[return to narrative text following Table 1]

The headline result is that at 1311 species *P. spumarius* has far more documented host plants than any other herbivorous insect (Table 2). They include ferns, herbs, shrubs, vines and trees, annuals and perennials, grasses and forbs, plants of the tropics, subtropics, temperate and boreal zones, conifers–just about every imaginable kind of vascular plant except those living submerged in aquatic environments. This fantastic species level diversity is reinforced in the higher order taxonomic diversity, 117 families and 631 genera. Table 3 summarizes the distribution of host species by family for all families represented by 10 or more species.

A large majority of host records, 1113 (84.9%), are for nymphs only. Eighty-eight (6.7%) are for nymphs and adults, and 6.6% (86) for adults alone (in 24 cases the life stage could not be determined or reasonably inferred from the information available). We include about 1890 geographical area records. Most hosts have been recorded from a single geographical area, but many are recorded from two or more. Multiple area hosts are typically widespread weedy herbs or common garden plants. Europe and North America account for most geographical records, 73.2% and 19.6% respectively, but Hawaii (4.1%) and New Zealand (2.8%) are well represented for their size. Britain and Ireland loom especially large, alone accounting for 37.2% of all records, in major part a reflection of the BRIGIT program. BRIGIT citizen science records include 358 hosts that are not duplicated in preexisting sources, a full 27.3% of all recorded hosts. Another 198 (15.1%) represent records that are new to Britain and Ireland. Seventy-two (5.9%) represent confirmation of hosts recorded for Britain and Ireland in preexisting sources. In total, BRIGIT citizen science records include 628 (47.9%) of the 1311 recorded *P. spumarius* hosts.

[continued after Tables 2 and 3]

Table 2. Selected "extreme polyphage" insects, including the insect species with the highest documented numbers of host plants, and, for comparison, a few notorious examples, such the Spongy moth and the Spotted lantern fly, as well as the second ranking spittlebug, *Aphrophora alni*. Note that *P. spumarius* has far more recorded hosts than any of the comparison species.

			**Number of host plants**			
**Insect species**	**Order & Family**	**Common name**	**Species**	**Genera**	**Families**	**Reference**
Philaenus spumarius (L.)	Hemiptera: Aphrophoridae	Meadow spittlebug	1311	631	117	present work*
Hyphantria cunea (Drury)	Lepidoptera: Arctiidae	Fall webworm	636	326	113	[201]*†
Coccus hesperidum L.	Hemiptera: Coccidae	Brown soft scale	552	417	138	[50]*
Epiphyas postvittana (Walker)	Lepidoptera: Tortricidae	Light brown apple moth	545	363	121	[202]
Aspidiotus nerii Bouche	Hemiptera: Diaspididae	Ivy scale	507	359	121	[50]*
Hemiberlesia lataniae (Signoret)	Hemiptera: Diaspididae	Palm scale	478	360	120	[50]*
Saissetia coffea (Walker)	Hemiptera: Coccidae	Hemispherical scale	412	313	112	[50]*
Lygus rugulipennis Poppius	Hemiptera: Miridae	European tarnished plant bug	402	226	57	[49]*
Lygus lineolaris (Palisot de Beauvois)	Hemiptera: Miridae	Tarnished plant bug	333	220	55	[203]*
Myzus persicae (Sulzer)	Hemiptera: Aphididae	Green peach aphid	305	–	72	[204]‡
Helicoverpa armigera (Hübner)	Lepidoptera: Noctuidae	Corn earworm	>300	–	68	[205]

Lymantria dispar (L.)	Lepidoptera: Erebidae	Spongy moth	>300	–	–	[206]
Aphis fabae Scopoli	Hemiptera: Aphididae	Blackbean aphid	293	–	71	[204]‡
Acherontia atropos (L.)	Lepidoptera: Sphingidae	African death's-head moth	208	102	40	[207]
Automeris postalbida Schaus	Lepidoptera: Saturniidae	N/A	188	125	59	[207]
Lycorma delicatula (White)	Hemiptera: Fulgoridae	Spotted lantern fly	172	100	33	[208]
Aphrophora alni (Fallén)	Hemiptera: Aphrophoridae	Alder spittlebug	145	89	38	VT, unpublished*

*Adjusted for generic redundancy.

†Includes some artificial cage feeding experiments.

‡UK hosts only

Table 3. *Philaenus spumarius* host plant families with 10 or more host species, ranked by number of host species. Also included are the number of genera for each host family, the percent of host species from that family among all host species, the cumulative percent of all host species going down the ranking, the number of species in each host family, and an index of the occurrence of host species by family weighted for family size. The weighted occurrence index is the number of host species in each family divided by the total number of species in each family and expressed as a percentage. It provides a crude measure of the relative prominence of each host family, taking into account the large differences in numbers of species per family.

Rank	Family	Number genera	Number ~~species~~	Percent species	Cumulative percent species	Number species in family[48]	Weighted occurrence in[illegible]
1	**Asteraceae**	104	222	16.9%	16.9%	24700	0.90
2	**Rosaceae**	31	110	8.4%	25.3%	2950	3.73
3	**Fabaceae**	33	76	5.8%	31.1%	19500	0.39
4	**Poaceae**	49	73	5.6%	36.7%	12000	0.61
5	**Lamiaceae**	24	62	4.7%	41.4%	7530	0.82
6	**Apiaceae**	35	50	3.8%	45.2%	3575	1.40
7	**Brassicaceae**	27	43	3.3%	48.5%	3628	1.19
8	**Caprifoliaceae**	16	34	2.6%	51.1%	825	4.12
9	**Caryophyllaceae**	11	32	2.4%	53.5%	2625	1.22
10	**Plantaginaceae**	6	27	2.1%	55.6%	1900	1.42
11	**Polygonaceae**	8	26	2.0%	57.6%	1200	2.17
12	**Ranunculaceae**	10	24	1.8%	59.4%	2346	1.02

13	**Onagraceae**	5	21	1.6%	61.0%	656	3.20
14	**Campanulaceae**	5	19	1.4%	62.5%	2300	0.83
15	**Ericaceae**	10	19	1.4%	63.9%	4250	0.45
16	**Primulaceae**	4	19	1.4%	65.4%	2790	0.68
17	**Boraginaceae**	12	18	1.4%	66.7%	2535	0.71
18	**Geraniaceae**	3	18	1.4%	68.1%	830	2.17
19	**Rubiaceae**	8	18	1.4%	69.5%	13620	0.13
20	**Asparagaceae**	11	13	1.0%	70.5%	2900	0.45
21	**Malvaceae**	8	13	1.0%	71.5%	4225	0.31
22	**Oleaceae**	7	13	1.0%	72.5%	790	1.65
23	**Betulaceae**	5	12	0.9%	73.4%	167	7.19
24	**Fagaceae**	3	11	0.8%	74.2%	927	1.19
25	**Papaveraceae**	6	11	0.8%	75.1%	775	1.42
26	**Pinaceae**	3	11	0.8%	75.9%	228	4.82
27	**Salicaceae**	2	11	0.8%	76.7%	1220	0.90
28	**Sapindaceae**	3	11	0.8%	77.6%	1860	0.59
29	**Solanaceae**	6	11	0.8%	78.4%	2600	0.42
30	**Amaryllidaceae**	2	10	0.8%	79.2%	1600	0.63
31	**Scrophulariaceae**	6	10	0.8%	79.9%	1830	0.55

*Number of *P. spumarius* host species in family as a percent of all species in family

Discussion

Philaenus spumarius appears to be the most polyphagous insect herbivore

~~We lead off with the horse race.~~ At 1311 species, *Philaenus spumarius* has, to our knowledge, more documented hosts than any other herbivorous insect. For comparison, Table 2 lists some of the serious contenders. Following Normark and Johnson [209], we limit comparisons to insects that feed directly on plant tissues. This excludes organisms like bees and syrphid flies that feed on pollen and nectar, flies that feed on rotting fruit, and leafcutter ants that attack plants but actually eat fungi that they cultivate on the plant material. None of the comparable insect species for which we have found data approach *P. spumarius* in number of host species. *Hyphantria cunea* (Drury), the fall webworm caterpillar weighs in closest, with a bit less than half the *P. spumarius* host numbers, though the webworm data include an unspecified number of artificial feeding tests [201], which we omitted in our compilation for *P. spumarius*. The closest arthropod competitor we have found is an arachnid, the red spider mite, *Tetranychus urticae* Koch, which is said to have more than 1100 hosts in over 140 plant families [210]. This puts *T. urticae* at the same order of magnitude as *P. spumarius* in host species number and substantially greater in host family number, the latter probably due to higher representation of plant families confined to the tropics.

Why is *P. spumarius* so polyphagous?

Our results substantiate Ossiannilsson's undocumented 1981 assertion that *P. spumarius* has more than 1000 hosts [211], ~~a guestimate~~ that had become embedded in the literature (cf.

[4,54,212]), despite lack of supporting evidence. A 1977 statement by Halkka & Mikkola [213] that there are “nearly 4000 recorded food-plant species” is clearly a typographical error. The documented 1311 species in 117 families put *P. spumarius* squarely in the category of “extreme polyphage”, defined by Normark & Johnson [209] as species that feed across more than 20 plant families. Like many extreme polyphages, it is a geographically widespread and invasive pest species, with very high population sizes. However, it does not exhibit other characteristics that Normark & Johnson [209] associate with extremely polyphagous insects, such as flightless females, larval dispersal, parthenogenesis or partiality to woody plants .

What characteristics have, in fact, contributed to the extraordinarily broad host range? Two factors are probably paramount. The first is xylem sap feeding, a nutritional mode that permits access to a food source that is similar across a wide range of host plants and not chemically defended (refs. in [214]). Xylem feeding apparently permits *P. spumarius* to feed on almost any plant it can penetrate with its mouth parts. The second factor is wide geographical range and the ability to thrive in climates from Hawaii, just south of the Tropic of Cancer [28], to within 65 km of the Arctic Circle in Finland [36]. Most or all extreme polyphages have cosmopolitan or invasive distributions [209].

Among xylem feeding insects, which include spittlebugs, cicadas and one subfamily of leafhoppers, *P. spumarius* is singular in its occupation of most of the Holarctic plus multiple distant islands. By that standard, other xylem feeders have been modest travelers. In addition to *P. spumarius*, four other spittlebug species have been introduced from Europe to North America [215], two others to Hawaii [216], and one other to New Zealand [217]. Two xylem feeding leafhoppers have been introduced from North America to Europe [218,219], and three cicada species have hopped from New Zealand’s North Island to South Island ([220] & C. Simon,

personal communication). None have achieved anything approaching the reach of *P. spumarius*. Perhaps not coincidentally, the other well-documented spittlebug extreme polyphage, *Aphrophora alni* (Table 2), is among the four other spittlebugs introduced from Europe to North America. It is not clear whether wide distribution is a cause or effect of extreme polyphagy. Each is clearly predisposed to promote the other [209].

Philaenus spumarius polyphagy seems to be a recent evolutionary development. It is one of a cluster of eight closely related *Philaenus* species living around the Mediterranean Basin [221]. Five are narrow monophages as nymphs, four feeding exclusively on the lily *Asphodelus ramosus* L. (or its close relatives *A. aestevus* or *A. microcarpus*) and one on *Eryngium* [222]. Two, *P. spumarius* and its very closely related sister species *Philaenus tesselatus*, are broad polyphages, though the extent of polyphagy is much less studied in *P. tesselatus* [223,224]. The host status of the eighth species, *Philaenus arslani*, is uncertain. It has been collected from a modest variety of hosts, including three thistles, *Cistus* and “diverse shrubs” [150], all apparently but not explicitly adult hosts.

Maryańska-Nadachowska et al. [221,225] propose that the line leading to *P. spumarius* originated from an *Asphodelus*-feeding ancestor between 7.9 and 3.7 Mya. If so, *P. spumarius* broke out of the Mediterranean monophage pack and spread to an enormous variety of hosts in a relatively short geological time period, exhibiting what Normark & Johnson [209] describe as a “niche explosion”. Why? One answer might be the evolution of a more extensive arsenal of gene families involved in digestion, detoxification and transport of xenobiotics, as suggested for the red spider mite [210], the only arthropod we have found with a comparable host range, and for the green peach aphid [226] and corn earworm [205], among the runners up for most

polyphagous insect herbivore (Table 2). On the other hand, the fact that xylem feeders encounter so few xenobiotics may make heroic detoxification capacity unnecessary. Ongoing work to sequence the complete *P. spumarius* genome [227] should provide data for a comparative analysis of the evolution of feeding versatility-related genes in relationship to niche explosion.

Whether or not accompanied by extensive changes in the food assimilation related genome, the rapid evolution of the of *P. spumarius* line from narrow monophagy to extreme polyphagy may have been facilitated by the feeding ecology of the adults. *Asphodelus* lilies die back in the Mediterranean summer dry season. *Philaenus* species dependent on *Asphodelus* as nymphs move to alternative hosts as adults [222], typically ectomycorrhizal trees and shrubs [228], a broadening of host range that may have set the stage for the evolution of polyphagy in the *P. spumarius* line. Although it has been stated that Mediterranean climate *P. spumarius* aestivate on these summer hosts [44] [222], there is no evidence for a state of summer torpor or hibernation ([69] & VT observations in California).

The apparent evolution of an extreme polyphage from monophagic ancestors in a relatively short evolutionary interval is highly unusual. Polyphages are rare among herbivorous insects ([229,230] and references therein), extreme polyphages even more so [209]. Had it been included in the most recent world survey of insect host plant breadth by Forister et al. [231], *Philaenus spumarius* would have been, in the most literal sense, off the charts. It seems to be one of a kind. It is also a clear counterexample to the suggestion that extreme polyphagy is an illusion based on multiple indistinguishable cryptic species feeding on different hosts [204,226]. Extensive work on mitochondrial haplotype distribution in *P. spumarius* rules out multiple unrecognized cryptic

species, although its mitochondrial lineages are bifurcated into two distinct clades [51,52,232] and one study suggested the presence of an unrecognized cryptic species in Anatolia and the Caucasus [233].

Patterns in host plant usage

Given that *P. spumarius* seems able and willing to feed on almost any available host, what patterns in host usage can we discern? In sheer species numbers (Table 3) the Asteraceae (222) win hands down, with over twice as many hosts as the runner up Rosaceae (110), followed by the Fabaceae (76) and the Poaceae (73). The latter two high ranking groups merit special comment. Spittlebugs have a demonstrated affinity for nitrogen-fixing hosts, including many Fabaceae [214] (Figure 1a). In *P. spumarius* this is reflected in its pest status legume forage crops in North America. Though it occurs on greater host species numbers in Asteraceae, it achieves highest densities on Fabaceae, up to 1280 nymphs/m^2 in on *M. sativa* [14]. The large numbers of Poaceae hosts (71) are surprising in the other direction. It has long been recognized that *P. spumarius* favors herbaceous dicots and is relatively rare on grasses [36]. While grasses as a group are not preferred hosts, the present results demonstrate that there are relatively large numbers of grass host species, which contribute markedly to total host diversity, and it is clear that *P. spumarius* is sometimes locally common on grasses. Booth [37], for example, found *P. spumarius* plentiful on grasses at some open sites in New Zealand and shaded sites in Wales, while Lester et al. [234] found *P. spumarius* to be relatively common on grasses during the Scottish professional BRIGIT survey.

At the high end of the host spectrum, *P. spumarius* is found not only on large numbers of Asteraceae species, but occurs in large numbers and high density on some individual species,

including several *Solidago* spp. and a number of thistles. Among the Rosaceae, *Filipendula ulmaria* by itself accounted for 22% of 40,737 nymphal host records collected by Halkka and his colleagues in Finland [213]. This highlights a major limitation of species lists as a measure of host diversity. They count occurrence but not frequency, though local frequency is often recorded in the underlying sources.

Another way to look at host attraction is to compare the ratio of *P. spumarius* hosts in a given family to the total number of species in that family. Table 3 includes a weighted occurrence index, the percentage of *P. spumarius* host species among all species in a plant family. This corrects, in a rough and ready way, for the fact that some plant families are small and some enormous. Among families with at least ten *P. spumarius* hosts, the index ranges from a low of 0.12 for Rubiaceae to a high of 7.19 for Betulaceae. In relation to total species numbers, *P. spumarius* occurs on a small proportion of Rubiaciae and a high proportion of Betulaceae. The Pinaceae (4.82), Caprifoliaceae (4.00), Rosaceae (3.42) and Onagraceae (3.05) are also high scoring. In contrast, two of the top three host families, Asteraceae (0.86) and Fabaceae (0.38), are knocked out of this competition by their enormous species numbers. The high rankings of two families comprised solely of trees and shrubs, Betulaceae and Pinaceae, might seem counterintuitive for an insect that clearly favors herbs, but both groups are ectomycorrhizal and their high scores are consistent with the general overrepresentation of this category among spittlebug hosts [228].

Plant morphology also clearly plays an important role in host plant selection. Early instar nymphs are especially attracted to plants with rosette form or other forms of growth that favor closely apposed leaf surfaces [5], no doubt because compact leafy clusters in close proximity to soil moisture form an advantageous early-instar nymphal microhabitat. Later instars tend to favor

tall and robust perennial herbs [213]. On the other hand, plant features like abundant trichomes and lignification of tissues clearly deter *P. spumarius* feeding [90,235]. When *P. spumarius* nymphs feed on woody plants it is invariably on new, unlignified growth, such as saplings, adventitious shoots of trees, or growing areas at the tips of branches ([5,121] and our observations).

Are there any otherwise apparently suitable plants on which *P. spumarius* does not feed? The most intriguing possibility is crownvetch, *Coronilla varia* L. (synonym *Securigera varia* (L.) Lassen), a Eurasian legume widely planted for forage and roadside erosion control in Eastern North America. Wheeler [236] reports that he found *P. spumarius* "in small numbers as adults only" on crownvetch but excludes it from his extensive list of arthropods collected on crownvetch in Pennsylvania. He adds that F.V. Grau, the founder of crownvetch studies in the USA, reported that he had never seen spittlebugs on this species in 28 years of work. We found no other records for crownvetch, a particularly unexpected result because *C. varia* is a nitrogen-fixing forage legume, the category of host on which *P. spumarius* otherwise reaches greatest densities in the USA. Apparently, something exceptional about its biology wards off *P. spumarius*. This makes it a promising candidate species for understory plantings in orchards and groves where there is a desire to suppress *P. spumarius* vector populations [237].

It also appears that *P. spumarius* nymphs may not occur on *Asclepias*, species of which are notoriously well-defended chemically. Beirne ([65] reports that *P. spumarius* nymphs do not feed on *Asclepias* species and the only *P. spumarius Asclepias* record we have found is for adults in Maryland (Table 1). There are, however, *Asclepias* nymphal records for two *Lepyronia* species [126,238], demonstrating that this genus is not off-limits to all spittlebugs. Schmidt [25] says that he never observed spittles on *Chenopodium* or *Atriplex*. *Chenopodium album*, the

species to which he is most likely referring, has been widely recorded as a *P. spumarius* host, both nymphal and adult, but *Atriplex* has only been observed as a host once, and only for adults (Table 1), suggesting that this genus may not be hospitable to nymphs. In general, our results sustain the early observations of Schmidt [25] and Fabre [83] that nymphs feed successfully on many plants that are chemically well-defended.

Although *P. spumarius* has been recorded on several fern species (Table 1), it has not been recorded on bryophytes. Press and Whittaker [239] illustrate a spittle of the grass-feeding spittlebug *Neophilaenus lineatus* on a moss (*Polytrichum commune*), demonstrating that mosses are within the realm of plausible hosts. Notable plant categories on which *P. spumarius* records are rare in relationship to their numbers are Orchidaceae, Bromeliaceae, and CAM plants as a group. This is not surprising for orchids and bromeliads, the large majority of which are tropical epiphytes, putting them largely out of the geographical and ecological range of *P. spumarius*. It is more surprising for CAM plants, many in the Crassulaceae (eight species in Table 1), which are diverse and widely distributed in areas and habitats that *P. spumarius* frequents. CAM plants (which overlap to include many species in the Orchidaceae and Bromeliaceae) maintain close control of daytime transpiration, perhaps interfering with the accessibility of xylem sap.

Implications for *Xylella fastidiosa* management

The most important lesson to be drawn from this review is that *P. spumarius* can and does feed on an extremely diverse array of plants, including, it appears, almost any vascular plant that comes its way with sufficiently accessible xylem vessels, the only apparent exceptions being, as noted above, *Coronilla varia* and *Asclepius* species. *Philaenus spumarius* nymphs are relatively sessile, moving infrequently, if at all, among hosts. By analogy with their leafhopper vector

counterparts [240], they ~~probably~~ lose any *X. fastidiosa* infection upon molting, which occurs five times during nymphal development. In consequence, nymphs are not effective vectors. In contrast, adults are effective vectors [110] and probably as versatile as nymphs in host range, though with many fewer hosts documented (Table 1). Although efficacy of transmission varies greatly with host species [1], *P. spumarius* adults are exceptionally well positioned to vector *X. fastidiosa* quickly and widely wherever the two co-occur, contingent on local conditions that favor the propagation of the bacterium within and among host plants [22,40,106]. Potential counter measures include: 1) local elimination or population reduction of *P. spumarius* by management of agriculturally adjacent host plants [2,106,212,237], 2) reduction or elimination of plant sources of *Xylella fastidiosa* infection [21], and 3) reduction of target plant susceptibility, through selection of cultivars with genetic resistance or other measures that reduce plant vulnerability to transmission and/or infection [2,21,241,242]. In turn, *P. spumarius* can be used as a sentinel organism to detect and monitor the presence of *X. fastidiosa* in local environments [243].

Going forward, we suggest several guidelines for future studies of *P. spumarius* on host plants. First and foremost, investigators should always specify life stage. A substantial number of past reports, especially from the agricultural sector, have omitted this important information. Second, to the degree possible, quantify the results. Counts are best, but even simple qualitative observations, such as “rare” or “abundant” are helpful, especially for observations on adults. Third, where *P. spumarius* nymphs are abundant, record the plants on which they are apparently absent, especially in instances in which the uninfested plant species are frequent and apparently suitable for feeding. This will assist in the ongoing search for alternative understory plants suitable for reducing *P. spumarius* numbers in agricultural settings. Recent screening of nymphs

in the Basilicata Region of Italy by Trotta et al. [11] is a model for this approach. The authors include data on 48 plant species that hosted nymphs *and* on 17 species that did not.

Lessons from the BRIGIT citizen scientist project

Mass-participation citizen science projects have a well-established history of contributing environmental and ecological data over numerical, spatial and temporal scales that would be impossible to collect by professional researchers alone [244,245]. Concerns about the quality of such data [246] are counterbalanced by recent studies indicating that the reliability of citizen science data can be significantly enhanced by training, data validation and ground-truthing [247,248].

The BRIGIT citizen science project proved to be a powerful tool for the rapid collection of large amounts of *P. spumarius* host plant usage data across the UK. Spittle was found to be a reliable focus for citizen science activity, being highly visible during the nymphal season and largely unmistakable for any other natural phenomena. At least in the UK, therefore, spittle on herbaceous dicots could be used for rapid assessment of both the presence and abundance of *P. spumarius*, as part of a future surveillance strategy for *X. fastidiosa*.

Finally, we note that the BRIGIT project is the latest installment in a long history of amateur contributions to *P. spumarius* host records, including the major works of Hugo Schmidt [26] [25], Ernest Noury [35] and Jennifer Owen [38]. In that sense, the work reported here is a monument to synergism between professional scientists and dedicated amateurs in the advancement of knowledge.

Acknowledgements

We dedicate this paper to our departed colleagues Olli Halkka and David Lees, and to John Whittaker, pioneers in evolutionary and ecological studies of *P. spumarius* and other spittlebugs. We thank librarians Mary Beth Riedner (Roosevelt University, retired), Gwen Short (Ohio State University) and Mai Reitmeyer (American Museum of Natural History) for tracking down some of the more obscure and hard-to-access references necessary for a review of this kind. Adeline Soulier-Perkins (Museum National d'Histoire Naturelle) provided a hard-to-find copy of Noury's host compendium [35]. Martin Harvey (UK Biological Records Centre), Kirsty Gamble (Leicestershire and Rutland Environmental Records Centre) and Alison Clague (Leicester County Council) helped us track down Jennifer Owen's archived host records. Chris Simon (University of Connecticut) provided unpublished information on New Zealand cicada introductions. Benjamin Normark (University of Massachusetts, Amherst) provided helpful insights and leads on extreme polyphages. Numerous colleagues over the years have shared unpublished host observations, a handful used here and all valuable to the host tracking effort. The BRIGIT project was funded by UK Research and Innovation through the Strategic Priorities Fund, by a grant from the Biotechnology and Biological Sciences Research Council, with support from the UK Department for Environment, Food and Rural Affairs and the Scottish Government, to AJAS and CH. We thank Saskia Hogenhout, Sam Mugford, Roberto Biello and Qun Liu (John Innes Centre, UK) for providing the worldwide host plant records collected during the BRIGIT project.

References

1. Bodino DN, Cavalieri DV, Dongiovanni DC, Saponari DM, Bosco PD. Bioecological traits of spittlebugs and their implications on the epidemiology and control of *Xylella fastidiosa* epidemic in Apulia (Southern Italy). Phytopathology. 2023. doi:10.1094/PHYTO-12-22-0460-IA

2. Cornara D, Bosco D, Fereres A. *Philaenus spumarius*: when an old acquaintance becomes a new threat to European agriculture. J Pest Sci. 2018;91: 957–972. doi:10.1007/s10340-018-0966-0

3. Sicard A, Zeilinger AR, Vanhove M, Schartel TE, Beal DJ, Daugherty MP, et al. *Xylella fastidiosa*: insights into an emerging plant pathogen. Annu Rev Phytopathol. 2018;56: 181–202. doi:10.1146/annurev-phyto-080417-045849

4. Trkulja V, Tomić A, Iličić R, Nožinić M, Milovanović TP. *Xylella fastidiosa* in Europe: from the introduction to the current status. Plant Pathol J. 2022;38: 551–571. doi:10.5423/PPJ.RW.09.2022.0127

5. Weaver CR, King DR. Meadow spittlebug, *Philaenus leucophthalmus* (L.). Research Bulletin 741. Ohio Agricutural Experiment Station, Wooster, Ohio; 1954.

6. Antonatos S, Papachristos DP, Kapantaidaki DE, Lytra IC, Varikou K, Evangelou VI, et al. Presence of Cicadomorpha in olive orchards of Greece with special reference to *Xylella fastidiosa* vectors. J Appl Entomol. 2021;144: 1–11.

7. Morente M, Cornara D, Plaza M, Durán JM, Capiscol C, Trillo R, et al. Distribution and relative abundance of insect vectors of *Xylella fastidiosa* in olive groves of the Iberian peninsula. Insects. 2018;9: 175. doi:10.3390/insects9040175

8. Dongiovanni C, Cavalieri V, Bodino N, Tauro D, Di Carolo M, Fumarola G, et al. Plant selection and population trend of spittlebug immatures (Hemiptera: Aphrophoridae) in olive groves of the Apulia region of Italy. J Econ Entomol. 2019;112: 67–74. doi:10.1093/jee/toy289

9. Latini A, Foxi C, Borfecchia F, Lentini A, De Cecco L, Iantosca D, et al. Tacking the vector of *Xylella fastidiosa*: geo-statistical analysis of long-term field observations on host plants influencing the distribution of *Phylaenus* [*sic*] *spumarius nymphs*. Environ Sci Pollut Res. 2019;26: 6503–6516. doi:10.1007/s11356-018-3870-5

10. Bodino N, Cavalieri V, Dongiovanni C, Saladini MA, Simonetto A, Volani S, et al. Spittlebugs of Mediterranean olive groves: host-plant exploitation throughout the year. Insects. 2020;11: 130. doi:10.3390/insects11020130

11. Trotta V, Forlano P, Caccavo V, Fanti P, Battaglia D. A survey of potential vectors of the plant pathogenic bacterium *Xylella fastidiosa* in the Basilicata Region, Italy. Bull Insectology. 2021;74: 273–283.

12. BRIGIT; Surveillance and response capacity for *Xylella fastidiosa*. In: John Innes Centre [Internet]. [cited 26 May 2023]. Available: https://www.jic.ac.uk/brigit/

13. Karban R, Strauss SY. Physiological tolerance, climate change, and a northward range shift in the spittlebug, *Philaenus spumarius*. Ecol Entomol. 2004;29: 251–254. doi:10.1111/j.1365-2311.2004.00576.x

14. Wiegert RG. Population energetics of meadow spittlebugs (*Philaenus spumarius* L.) as affected by migration and habitat. Ecol Monogr. 1964;34: 218–241.

15. Whittaker JB. Studies on the Auchenorrhyncha (Homoptera–Insecta) of Pennine moorland with special reference to the Cercopidae. PhD Thesis, Durham University. 1963.

16. Masters GJ, Brown VK, Clarke IP, Whittaker JB, Hollier JA. Direct and indirect effects of climate change on insect herbivores: Auchenorrhyncha (Homoptera). Ecol Entomol. 1998;23: 45–52. doi:10.1046/j.1365-2311.1998.00109.x

17. Stewart AJA, Lees DR. The colour/pattern polymorphism of *Philaenus spumarius* (L.) (Homoptera: Cercopidae) in England and Wales. Philos Trans R Soc Lond B Biol Sci. 1996;351: 69–89. doi:10.1098/rstb.1996.0005

18. Thompson V. Distributional evidence for thermal melanic color forms in *Philaenus spumarius*, the polymorphic spittlebug. Am Midl Nat. 1984; 288–295.

19. Stewart AJA, Lees DR. Genetic control of colour/pattern polymorphism in British populations of the spittlebug *Philaenus spumarius* (L.) (Homoptera: Aphrophoridae). Biol J Linn Soc. 1988;34: 57–79. doi:10.1111/j.1095-8312.1988.tb01948.x

20. Thompson V. Parallel colour form distributions in European and North American populations of the spittlebug *Philaenus spumarius* (L.). J Biogeogr. 1988; 507–512.

21. Saponari M, Giampetruzzi A, Loconsole G, Boscia D, Saldarelli P. *Xylella fastidiosa* in olive in Apulia: Where we stand. Phytopathology. 2019;109: 175–186. doi:10.1094/PHYTO-08-18-0319-FI

22. Olmo D, Nieto A, Borràs D, Montesinos M, Adrover F, Pascual A, et al. Landscape epidemiology of *Xylella fastidiosa* in the Balearic Islands. Agronomy. 2021;11: 473. doi:10.3390/agronomy11030473

23. Moralejo E, Gomila M, Montesinos M, Borràs D, Pascual A, Nieto A, et al. Phylogenetic inference enables reconstruction of a long-overlooked outbreak of almond leaf scorch disease (*Xylella fastidiosa*) in Europe. Commun Biol. 2020;3: 1–13. doi:10.1038/s42003-020-01284-7

24. EFSA EFS, Delbianco A, Gibin D, Pasinato L, Boscia D, Morelli M. Update of the *Xylella* spp. host plant database – systematic literature search up to 31 December 2021. EFSA J. 2022;20: e07356. doi:10.2903/j.efsa.2022.7356

25. Schmidt H. Weitere Bemerkungen zu “die Larve der Schaumzikade (*Aphrophora spumaria* L.) als gallenbildendes Tier.” Prometheus Leipz. 1914;26: 90–92.

26. Schmidt H. Die Larve der Schaumzikade (*Aphrophora spumaria* L.) als gallenbildendes Tier. Prometheus Leipz. 1914;25: 250–252.

27. Davis CJ. Additional hosts of *Philaenus spumarius* (L.). Proc Hawaii Entomol Soc. 1947;13: 30–31.

28. Davis CJ, Mitchell AL. Host records of *Philaenus spumarius* (Linn.) at Kilauea, Hawaii National Park (Homoptera: Cercopidae). Proc Hawaii Entomol Soc. 1946;12.

29. DeLong DM, Severin HHP. Spittle-insect vectors of Pierce’s disease virus. I. Characters, distribution, and food plants. Hilgardia. 1950;19: 339–356.

30. King DR. Ecology of the Meadow Spittlebug *Philaenus leucophthalmus* (L.); Family Cercopidae. PhD thesis, The Ohio State University. 1952.

31. Ahmed DD. Life History and Control of the Meadow Spittlebug *Philaenus leucophthalmus* (Linn.)(Homoptera, Cercopidae). PhD thesis, The Ohio State University. 1949.

32. Teller LW. The Meadow Spittlebug, *Philaenus leucophthalmus* (L.) in Maryland. PhD Thesis, University of Maryland. 1951.

33. Marshall DS. The control of the meadow spittlebug (*Philaenus leucophthalmus* (L.)). PhD Thesis, Cornell University. 1951.

34. Metcalf ZP. General Catalogue of the Homoptera. Fascicle VII. Cercopoidea. Part 3. Aphrophoridae. Waverly Press, Baltimore; 1962.

35. Noury EM. Les plantes-hôtes de la cicadelle *Philaenus spumarius* (L.). Cah Nat Bull Nat Paris Ns. 1965;21: 93–97.

36. Halkka O, Raatikainen M, Vasarainen A, Heinonen L. Ecology and ecological genetics of *Philaenus spumarius* (L.) (Homoptera). Ann Zool Fenn. 1967;4: 1–18.

37. Booth WJ. Aspects of Host Plant Relationships in Cercopidae (Homoptera: Auchenorrhyncha). PhD thesis, University of Wales, College of Cardiff. 1993.

38. Owen J. Wildlife of a Garden: A Thirty-Year Study. Royal Horticultural Society; 2010.

39. Owen DF. (1988) Native and alien plants in the diet of *Philaenus spumarius* (L.) (Homoptera: Cercopidae). Entomol Gaz. 1988;39: 327-328.

40. Bodino N, Demichelis S, Simonetto A, Volani S, Saladini MA, Gilioli G, et al. Phenology, seasonal abundance, and host-plant association of spittlebugs (Hemiptera: Aphrophoridae) in vineyards of northwestern Italy. Insects. 2021;12: 1012. doi:10.3390/insects12111012

41. Osborn H. Studies of Life Histories of Froghoppers of Maine. Bulletin 254. Maine Agricultural Experiment Station; 1916.

42. Krauss NLH. *Philaenus spumarius* (Linn.). Proc Hawaii Entomol Soc. 1945;12: 220.

43. Licent PÉ. Recherches d'anatomie et de physiologie comparées sur le tube digestif des homoptères supérieurs. La Cellule. 1912;28: 1–161.

44. Drosopoulos S. New data on the nature and origin of colour polymorphism in the spittlebug genus *Philaenus* (Hemiptera: Aphrophoridae). Ann Société Entomol Fr NS. 2003;39: 31–42. doi:10.1080/00379271.2003.10697360

45. Cornara D, Panzarino O, Santoiemma G, Bodino N, Loverre P, Mastronardi MG, et al. Natural areas as reservoir of candidate vectors of *Xylella fastidiosa*. Bull Insectology. 2021;74: 173–180.

46. ITIS. Integrated Taxonomic Information System. www.itis.gov. 2023. doi:10.5066/F7KH0KBK

47. PoWO. Plants of the World Online. http://www.plantsoftheworldonline.org/. 2023.

48. Christenhusz MJM, Byng JW. The number of known plants species in the world and its annual increase. Phytotaxa. 2016;261: 201–217. doi:10.11646/phytotaxa.261.3.1

49. Holopainen JK, Varis A-L. Host plants of the European tarnished plant bug *Lygus rugulipennis* Poppius (Het., Miridae). J Appl Entomol. 1991;111: 484–498. doi:10.1111/j.1439-0418.1991.tb00351.x

50. García Morales M, Denno BD, Miller DR, Miller GL, Ben-Dov Y, Hardy NB. ScaleNet: A literature-based model of scale insect biology and systematics. Database. doi: 10.1093/database/bav118. http://scalenet.info. 2016.

51. Seabra SG, Rodrigues ASB, Silva SE, Neto AC, Pina-Martins F, Marabuto E, et al. Population structure, adaptation and divergence of the meadow spittlebug, *Philaenus spumarius* (Hemiptera,

Aphrophoridae), revealed by genomic and morphological data. PeerJ. 2021;9: e11425. doi:10.7717/peerj.11425

52. Rodrigues AS, Silva SE, Marabuto E, Silva DN, Wilson MR, Thompson V, et al. New mitochondrial and nuclear evidences support recent demographic expansion and an atypical phylogeographic pattern in the spittlebug *Philaenus spumarius* (Hemiptera, Aphrophoridae). PLoS One. 2014;9: e98375.

53. Borges PAV, Gaspar C, Crespo LCF, Rigal F, Cardoso P, Pereira F, et al. New records and detailed distribution and abundance of selected arthropod species collected between 1999 and 2011 in Azorean native forests. Biodivers Data J. 2016; e10948. doi:10.3897/BDJ.4.e10948

54. Thompson V. Spittlebugs. Ed. by W.L. Lamp et al. Handbook of Forage and Rangeland Insects. Lanham, Maryland: Entomological Society of America; 2007. pp. 91–95.

55. Novikov DV, Novikova NV, Anufriev GA, Dietrich CH. Auchenorrhyncha (Hemiptera) of Kyrgyz grasslands. Russ Entomol J. 2006;15: 303–310.

56. Kozhevnikova AG. Monitoring of sucking pests of vegetable crops from the (Auchenorrhyncha) series of Uzbekistan. Am J Agric Biomed Eng. 2021;3: 1–5. doi:10.37547/tajabe/Volume03Issue04-01

57. Quilici S, Reynaud B, Bonfils J. Sur deux espèces d'Hémiptères Auchénorrhynques, nouvelles pour la Réunion et d'importance économique potentielle. Bull Société Entomol Fr. 1998 [cited 26 Oct 2022]. Available: https://agritrop.cirad.fr/401125/

58. Putman WL. Notes on the bionomics of some Ontario cercopids (Homoptera). Can Entomol. 1953;85: 244–248. doi:10.4039/Ent85244-7

59. Thompson V. A new spittlebug of the genus *Aphrophora* Germar, 1821 (Hemiptera: Cercopoidea: Aphrophoridae) abundant on invasive iceplant, *Carpobrotus edulis* (L.) N. E. Brown (Aizoaceae), in coastal California. Pan-Pac Entomol. 2021;97: 105–128. doi:10.3956/2021-97.3.105

60. Rodríguez J, Thompson V, Rubido-Bará M, Cordero-Rivera A, González L. Herbivore accumulation on invasive alien plants increases the distribution range of generalist herbivorous insects and supports proliferation of non-native insect pests. Biol Invasions. 2019;21: 1511–1527. doi:10.1007/s10530-019-01913-1

61. Tothova M, Toth P, Cagáň Ľ. Leafhoppers, planthoppers, froghoppers and cixiids (Auchenorrhyncha) on pigweeds as vectors of plant diseases. Acta Fytotech Zootech. 2004;7: 322–326.

62. Yildirim E, Özbek H. Insect fauna of sugar beet in sugar beet growing areas of Erzurum Sugar Factory. Proceedings of the Second Turkish National Congress of Entomology. Adana, Turkey; 1992. pp. 621–635.

63. Whittaker JB. Polymorphism of *Philaenus spumarius* (L.) (Homoptera, Cercopidae) in England. J Anim Ecol. 1968;37: 99–111. doi:10.2307/2713

64. Thanou ZN, Kontogiannis EG, Tsagkarakis AE. Impact of weeds on Auchenorrhyncha incidence and species richness in citrus orchards. Phytoparasitica. 2021;49: 333–347. doi:10.1007/s12600-020-00857-w

65. Beirne BP. Pest Insects of Annual Crop Plants in Canada: IV. Hemiptera-Homoptera, V. Orthoptera, VI. Other Groups. Entomological Society of Canada; 1972.

66. Blanton FS. Leafhoppers and Homoptera of related families collected in and adjacent to narcissus plantings.1937;30:972. J Econ Entomol. 1937;30: 972.

67. Löcker H. Arborikole Zikaden-Gilden in Slowenien:(Hemiptera, Auchenorrhyncha). Cicadina. 2003;6: 7–38.

68. Bodino N, Cavalieri V, Dongiovanni C, Plazio E, Saladini MA, Volani S, et al. Phenology, seasonal abundance and stage-structure of spittlebug (Hemiptera: Aphrophoridae) populations in olive groves in Italy. Sci Rep. 2019;9: 17725. doi:10.1038/s41598-019-54279-8

69. Albre J, Carrasco JMG, Gibernau M. Ecology of the meadow spittlebug *Philaenus spumarius* in the Ajaccio region (Corsica) – I: Spring. Bull Entomol Res. 2021;111: 246–256. doi:10.1017/S0007485320000711

70. Larivière M-C, Fletcher MJ, Larochelle A. Auchenorrhyncha (Insecta: Hemiptera): catalogue. Fauna N Z. 2010;63. doi:10.7931/J2/FNZ.63

71. Kaygin AT, Sönmezyildiz H, Ulgentürk S, Ozdemir I. Insect species damage on ornamental plants and saplings of Bartin Province and its vicinity in the western Black Sea region of Turkey. Int J Mol Sci. 2008;9: 526–541. doi:10.3390/ijms9040526

72. Nickel H. The Leafhoppers and Planthoppers of Germany (Hemiptera, Auchenorrhyncha): Patterns and Strategies in a Highly Diverse Group of Phytophagous Insects. Sofia: Pensoft Publishers,; 2003.

73. Malykh YN, Krisch B, Gerardy-Schahn R, Lapina EB, Shaw L, Schauer R. The presence of N-acetylneuraminic acid in Malpighian tubules of larvae of the cicada *Philaenus spumarius*. Glycoconj J. 1999;16: 731–739. doi:10.1023/A:1007115627708

74. Lodos N, Kalkandelen A. Preliminary list of Auchenorrhyncha with notes on distribution and importance of species in Turkey. VI. Families Cercopidae and Membracidae. Türkiye Bitki Koruma Derg. 1981;5: 133–149.

75. Kaygın AT, EkİcĠ B. Host diversity of *Philaenus spumarius* (L.) (Hemiptera: Cercopidae) in Bartın Region. Türkiye Entomoloji Bül. 2017;7: 7–13.

76. Archibald RD, Cox JM, Deitz LL. New records of plant pests in New Zealand. N Z J Agric Res. 1979;22: 201–207. doi:10.1080/00288233.1979.10420862

77. Dáder B, Viñuela E, Moreno A, Plaza M, Garzo E, del Estal P, et al. Sulfoxaflor and natural pyrethrin with piperonyl butoxide are effective alternatives to neonicotinoids against juveniles of *Philaenus spumarius*, the European vector of *Xylella fastidiosa*. Insects. 2019;10: 225. doi:10.3390/insects10080225

78. López-Mercadal J, Delgado S, Mercadal P, Seguí G, Lalucat J, Busquets A, et al. Collection of data and information in Balearic Islands on biology of vectors and potential vectors of *Xylella fastidiosa*. EFSA Support Publ EN-6925. 2021;18: 1–136. doi:10.2903/sp.efsa.2021.EN-6925

79. Karban R, Huntzinger M. Spatial and temporal refugia for an insect population declining due to climate change - ProQuest. 2021;12: 1–9.

80. Arbour G. A spittlebug in the lovage plant (*Philaenus spumarius*). In: Giles Arbour Bilingual Blog - Français/English [Internet]. 28 Jun 2009 [cited 18 Oct 2022]. Available: https://gillesarbour.wordpress.com/2009/06/28/a-spittlebug-in-the-lovage-plant-philaenus-spumarius/

81. Palin MA. *Ligusticum scoticum* L. (*Haloscias scoticum* (L.)). J Ecol. 1988;76: 889–902. doi:10.2307/2260580

82. Regnier R. Les Cicadelles écumantes de Normandie. Contribution à l'étude de *Ptyelus graminis* De Geer (Homoptere) et de ses variations. Bull Société Sci Nat Roue. 1936;70–71: 91–99.

83. Fabre J-H. La cicadelle écumeuse. Souvenirs Entomologiques (Septième Série), Études sur L'instinct et les Moeurs des Insectes. Paris: Libraire Delagrave; 1923. pp. 239–254.

84. Goetzke HH, Pattrick JG, Federle W. Froghoppers jump from smooth plant surfaces by piercing them with sharp spines. Proc Natl Acad Sci. 2019;116: 3012–3017. doi:10.1073/pnas.1814183116

85. Georghiou GP. The insects and mites of Cyprus. With emphasis on species of economic importance to agriculture, forestry, man and domestic animals. Athens, Greece: Kiphissia; 1977.

86. Yurtsever S. Inheritance of three dorsal color/pattern morphs in some Turkish *Philaenus spumarius* (Homoptera: Cercopidae) populations. Isr J Zool. 1999;45: 361–369. doi:10.1080/00212210.1999.10689005

87. Wood ZM, Jones PL. The effects of host plant species and plant quality on growth and development in the meadow spittlebug (*Philaenus spumarius*) on Kent Island in the Bay of Fundy. Northeast Nat. 2020;27: 168–185. doi:10.1656/045.027.0114

88. Nickel B. Untersuchungen über die Ernährung und die Aussscheidungen einheimischer Cercopidenlarven. PhD thesis, Technische Universität, München. 1979.

89. Kiss B, Rédei D, Koczor S. Occurrence and feeding of hemipterans on common ragweed (*Ambrosia artemisiifolia*) in Hungary. Bull Insectology. 2008;61: 195–196.

90. Hoffman GD, McEvoy PB. Mechanical limitations on feeding by meadow spittlebugs *Philaenus spumarius* (Homoptera: Cercopidae) on wild and cultivated host plants. Ecol Entomol. 1985b;10: 415–426. doi:10.1111/j.1365-2311.1985.tb00739.x

91. Fagan WF. Omnivory as a stabilizing feature of natural communities. Am Nat. 1997;150: 554–567.

92. Villa M, Rodrigues I, Baptista P, Fereres A, Pereira JA. Populations and host/non-host plants of spittlebugs nymphs in olive orchards from northeastern Portugal. Insects. 2020;11: 720. doi:10.3390/insects11100720

93. Yurtsever S, Bakırcıoglu D, Karahalıl B. A preliminary study on the metal content of the spittle of two cercopoid insects (Hemiptera: Cercopoidea, Aphrophoridae). Entomol Mon Mag. 2012;148: 95–108.

94. Kambo DS. Differences in Performance and Herbivory Along a Latitudinal Gradient for Common Burdock (*Arctium minus*). MS thesis, University of Toronto. 2012. Available: https://www.proquest.com/openview/3d724f30fec600cc04b26059562a7bff/1?pq-origsite=gscholar&cbl=18750

95. Rodríguez J, Cordero-Rivera A, González L. Impacts of the invasive plant *Carpobrotus edulis* on herbivore communities on the Iberian Peninsula. Biol Invasions. 2021;23: 1425–1441.

96. Rösch V, Schmitz G. Phytophagous arthropod fauna of Chinese Mugwort *Artemisia verlotiorum*, Lamotte, 1877 (Asteraceae) in Central Europe, particularly the Lake Constance region. Entomol Gen. 2014;35: 33–45.

97. Zeybekoğlu Ü, Turgut F. A study on the life cycle and population density of *Philaenus spumarius* (L.) (Hom: Cercopidae) in Samsun. Ondokuz Mayıs Üniversitesi Ziraat Fakültesi Derg Anadolu Tarım Bilim Derg. 2003;18: 49–54.

98. Sevarika M, Rondoni G, Ganassi S, Pistillo OM, Germinara GS, De Cristofaro A, et al. Behavioural and electrophysiological responses of *Philaenus spumarius* to odours from conspecifics. Sci Rep. 2022;12: 8402. doi:10.1038/s41598-022-11885-3

99. Grant JF, Lambdin PL, Follum RA. Infestation levels and seasonal incidence of the meadow spittlebug (Homoptera: Cercopidae) on musk thistle in Tennessee. J Agric Entomol USA. 1998;15: 83–91.

100. Batra SWT, Coulson JR, Dunn PH, Boldt PE. Insects and Fungi Associated with *Carduus* Thistles (Compositae). U.S. Department of Agriculture, Technical Bulletin No. 1616. 1981.

101. Johnson JB, McCaffrey JP, Merickel FW. Endemic phytophagous insects associated with yellow starthistle in northern Idaho. Pan-Pac Entomol. 1992;68: 169–173.

102. Henderson G, Hoffman GD, Jeanne RL. Predation on cercopids and material use of the spittle in aphid-tent construction by prairie ants. Psyche (Stuttg). 1990;97: 43–53. doi:10.1155/1990/28269

103. Batters J. The Impact of Invasive Thistle Species on Dynamics of Cercopidae in the Stikine-Skeena Region of Northern British Columbia. Undergraduate honors thesis, Lakehead University. 2020.

104. Lavigne R. Biology of *Philaenus leucophthalmus* (L.), In Massachusetts1. J Econ Entomol. 1959;52: 904–907. doi:10.1093/jee/52.5.904

105. Ochoa J. MJ. Cultivo de cardo para la producción de biomasa. Surcos Aragón. 2000; 41–43.

106. Mesmin X, Chartois M, Borgomano S, Rasplus J-Y, Rossi J-P, Cruaud A. Interaction networks between spittlebugs and vegetation types in and around olive and clementine groves of Corsica; implications for the spread of *Xylella fastidiosa*. Agric Ecosyst Environ. 2022;334: 107979. doi:10.1016/j.agee.2022.107979

107. Weaver CR. Improvement in hay yields resulting from control of the meadow spittlebug. J Econ Entomol. 1950;43.

108. Karban R. Interspecific competition between folivorous insects on *Erigeron glaucus*. Ecology. 1986;67: 1063–1072. doi:10.2307/1939829

109. Batra SWT. Insects associated with weeds of the northeastern United States: Quickweeds, *Galinsoga ciliata* and *Galinsoga parviflora* (Compositae). Enviromental Entomol. 1979;8: 1078–1082. doi:10.1093/ee/8.6.1078

110. Cornara D, Sicard A, Zeilinger AR, Porcelli F, Purcell AH, Almeida RPP. Transmission of *Xylella fastidiosa* to grapevine by the meadow spittlebug. Phytopathology. 2016;106: 1285–1290. doi:10.1094/PHYTO-05-16-0202-R

111. Randall J. Element stewardship abstract for *Hemizonia pungens* Torr. & Grey. The Nature Conservancy; undated. Available: https://www.invasive.org/gist/esadocs/documnts/hemipun.rtf

112. Syrett P, Smith LA. The insect fauna of four weedy *Hieracium* (Asteraceae) species in New Zealand. N Z J Zool. 1998;25: 73–83.

113. Zeleny J, Havelka J, Slama K. Hormonally mediated insect-plant relationships: Arthropod populations associated with ecdysteroid-containing plant, *Leuzea carthamoides* (Asteraceae). Eur J Entomol. 1997;94: 183–198.

114. Hambäck PA. Direct and indirect effects of herbivory: Feeding by spittlebugs affects pollinator visitation rates and seedset of *Rudbeckia hirta*. Écoscience. 2001;8: 45–50. doi:10.1080/11956860.2001.11682629

115. Hardy J. Notes on cuckoo-flowers and the cuckoo spit. Edinb New Philos J. 1862;16 (New Series): 70–76.

116. Markheiser A, Cornara D, Fereres A, Maixner M. Analysis of vector behavior as a tool to predict *Xylella fastidiosa* patterns of spread. Entomol Gen. 2020;40: 1–13. doi:10.1127/entomologia/2019/0841

117. Jobin A, Schaffner U, Nentwig W. The structure of phytophagous insect fauna on the introduced weed *Solidago altissima* in Switzerland. Entomol Exp Appl. 1996;79: 33–42. doi:10.1007/BF00333819

118. Wise MJ, Kieffer DL, Abrahamson WG. Costs and benefits of gregarious feeding in the meadow spittlebug, *Philaenus spumarius*. Ecol Entomol. 2006;31: 548–555. doi:10.1111/j.1365-2311.2006.00814.x

119. Buchele DE, Baskin JM, Baskin CC. Ecology of the endangered species *Solidago shortii*. iii. Seed germination ecology. Bull Torrey Bot Club. 1991;118: 288–291. doi:10.2307/2996643

120. Rodman GH. The story of the cuckoo spit. Proc Photomicrogr Soc. 1921;10: 31–42.

121. Ossiannilsson F. On the identity of *Cicada spumaria* Linneaus (1758) (Hemiptera. Homoptera), with notes on the breeding-plants of three Swedish cercopids. Opusc Entomol. 1950;15: 145–156.

122. Varty IW. A survey of the sucking insects of the birches in the maritime provinces. Can Entomol. 1963;95: 1097–1106. doi:10.4039/Ent951097-10

123. Mazzoni V. Contribution to the knowledge of the Auchenorrhyncha (Hemiptera Fulgoromorpha and Cicadomorpha) of Tuscany (Italy). Redia. 2005;88: 85–102.

124. Wahlgren E. Wahlgren, E. 1944. Cecidiologiska anteckningar. Entomol Tidskr. 65: 50–121.

125. Yates CN, Murphy SD. Observations of herbivore attack on garlic mustard (*Alliaria petiolata*) in Southwestern Ontario, Canada. Biol Invasions. 2008;10: 757–760. doi:10.1007/s10530-007-9169-y

126. Doering KC. Host plant records of Cercopidae in North America, North of Mexico (Homoptera). J Kans Entomol Soc. 1942;15: 65–72.

127. Zimmerman EC. Insects of Hawaii. Vol 4. Homoptera: Auchenorrhyncha. Honolulu: University of Hawaii Press; 1948.

128. McPartland JM, Clarke RC, Watson DP. Hemp Diseases and Pests: Management and Biological Control. CABI; 2000.

129. Waipara NW, Winks CJ, Smith LA, Wilkie JP. Natural enemies of Japanese honeysuckle *Lonicera japonica* in New Zealand. N Z Plant Prot. 2007;60: 158–163. doi:10.30843/nzpp.2007.60.4675

130. Batra SWT. Insects associated with weeds in the northeastern United States. iii. Chickweed, *Stellaria media*, and stitchwort, *S. graminea* (Caryophyllaceae). J N Y Entomol Soc. 1979;87: 223–235.

131. Nencioni A, Pastorelli R, Bigiotti G, Cucu MA, Sacchetti P. Diversity of the bacterial community associated with hindgut, Malpighian tubules, and foam of nymphs of two spittlebug species (Hemiptera: Aphrophoridae). Microorganisms. 2023;11: 466. doi:10.3390/microorganisms11020466

132. Hamilton KGA, Morales CF. Cercopidae (Insecta: Homoptera). Fauna N Z. 1992;25. doi:10.7931/J2/FNZ.25

133. Ward LK. The conservation of juniper: The associated fauna with special reference to southern England. J Appl Ecol. 1977;14: 81–120. doi:10.2307/2401829

134. Halkka O, Raatikainen M, Halkka L, Raatikainen T. Coexistence of four species of spittle-producing Homoptera. Ann Zool Fenn. 1977;14: 228–231.

135. Frey-Gessner E. Beitrag zur Hemiptern-Fauna des Ober-Wallis. Mitteilungen Schweiz Entomol Ges. 1862;1: 24–31.

136. Hartley SE, Gardner SM. The response of *Philaenus spumarius* (Homoptera: Cercopidae) to fertilizing and shading its moorland host-plant (*Calluna vulgaris*). Ecol Entomol. 1995;20: 396–399. doi:10.1111/j.1365-2311.1995.tb00474.x

137. Nixon D, Okely EF, Blackith RM. The distribution and morphometrics of spittle bugs on Irish blanket bog. Proc R Ir Acad B. 1975;75: 305–315.

138. Silva L, Tavares J. Phytophagous insects associated with endemic, Macaronesian, and exotic plants in the Azores. Avances en entomología ibérica. Museo Nacional de Ciencias Naturales (CSIC) y Universidad Autónoma de Madrid; 1995. pp. 179–188. Available: https://repositorio.uac.pt/handle/10400.3/789

139. Nunn ML, Hillier NK. Biodiversity and sweep sampling of selected leafhopper and beetle species in wild blueberries. J Acadian Entomol Soc. 2015;11: 17–21.

140. Maurice C, Bédard C, Fitzpatrick SM, Troubridge J, Henderson D. Integrated Pest Management for Cranberries in Western Canada, Technical Report #163. Agriculture and Agri-food Canada; 2000.

141. Whitfield GH, Ellis CR. The pest status of foliar insects on soybeans and white beans in Ontario. Proc Entomol Soc Ont. 1976;107: 47–55.

142. Mackun IR, Baker BS. Insect populations and feeding damage among birdsfoot trefoil–grass mixtures under different cutting schedules. J Econ Entomol. 1990;83: 260–267. doi:10.1093/jee/83.1.260

143. Ganassi S, Cascone P, Domenico CD, Pistillo M, Formisano G, Giorgini M, et al. Electrophysiological and behavioural response of *Philaenus spumarius* to essential oils and aromatic plants. Sci Rep. 2020;10: 3114. doi:10.1038/s41598-020-59835-1

144. Germinara GS, Ganassi S, Pistillo MO, Di Domenico C, De Cristofaro A, Di Palma AM. Antennal olfactory responses of adult meadow spittlebug, *Philaenus spumarius*, to volatile organic compounds (VOCs). PloS One. 2017;12: e0190454. doi:10.1371/journal.pone.0190454

145. Macklin PR. Spittle insects as food of the red-winged blackbird. The Auk. 1958;75: 225–225. doi:10.2307/4081906

146. Rekach VN, Dobretzova TA. A survey of insects injurious to utility and forage crops in Transcaucasia. 1935;45? x-42-y.

147. Everly RT. Evaluation of population estimates and rate of loss of forage for the meadow spittlebug, *Philaenus leucophthalmus*. Proc Indiana Acad Sci. 1958;68: 171–185.

148. Pearson WD. Effect of meadow spittlebug and Australian crop mirid on white clover seed production in small cages. N Z J Agric Res. 1991;34: 439–444. doi:10.1080/00288233.1991.10417688

149. Farnham NGJ. The polymorphism of *Philaenus spumarius*. Bull Amat Entomol Soc. 1983;42: 177–188.

150. Abdul-Nour H, Lahoud L. Révision du genre Philaenus Stål, 1864 au Liban, avec la description d’une nouvelle espèce *P. arslani*, n. sp. (Homoptera Auchenorrhyncha, Cercopoidea). Nouv Rev Entomol. 1995;12: 297–303.

151. Nixon P, McPherson J. An annotated list of phytophagous insects collected on immature black walnut trees in southern Illinois. Gt Lakes Entomol. 2017;10. Available: https://scholar.valpo.edu/tgle/vol10/iss4/8

152. Güçlü Ş, Hayat R, Özbek H. An investigation on phytophagous insect species on walnut (*Juglans regia* Linnaeus) in Erzurum and its neighbouring provinces. Turk J Entomol. 1995;19.

153. Reclaire A. Naamlijst der in Nederland en het aangrenzende gebied waargenomen Cicaden (Hemiptera-Homoptera). Entomol Ber. 11: 221–256.

154. Metin F, Zeybekoğlu Ü, Karaca İ. Insects on lavender in Isparta province, Turkey. Int J Agric Environ Food Sci. 2020;4: 425–431. doi:10.31015/jaefs.2020.4.5

155. Neubauer S, Kral J, Klimes K. Insect pests of peppermint. Nase Liecive Rastl. 1974;11: 38–41.

156. Dunn AJ. *Stachys germanica* L. J Ecol. 1997;85: 531–539. doi:10.2307/2960575

157. Jürisoo V. Agro-Ecological Studies on Leafhoppers (Auchenorrhyncha, Homoptera) and Bugs (Heteroptera) at Ekensgård Farm in the Province of Hälsingland, Sweden. PhD thesis, Stockholm University. 1964. Available: http://urn.kb.se/resolve?urn=urn:nbn:se:su:diva-168741

158. Ellis D. Biological Control of Purple Loosestrife in Connecticut. Purdue University; 1997. Available: http://www.ceris.purdue.edu/napis/states/ct/etpls/pls1997.html

159. Kaçar G, Başpınar H, Zeybekoğlu Ü, Ulusoy MR. Potential treat for olives: *Philaenus spumarius* (Linnaeus) (Cercopidae) and Auchenorrhyncha species (Hemiptera). 2017;57: 463–471. doi:10.16955/bitkorb.334878

160. MacGarvin M. Species-area relationships of insects on host plants: Herbivores on rosebay willowherb. J Anim Ecol. 1982;51: 207–223. doi:10.2307/4320

161. Puentes A, Johnson MTJ. Tolerance to deer herbivory and resistance to insect herbivores in the common evening primrose (*Oenothera biennis*). J Evol Biol. 2016;29: 86–97. doi:10.1111/jeb.12764

162. Ivey CT, Carr DE. Effects of herbivory and inbreeding on the pollinators and mating system of *Mimulus guttatus* (Phrymaceae). Am J Bot. 2005;92: 1641–1649. doi:10.3732/ajb.92.10.1641

163. Johnson WT, Lyon HH. Insects That Feed on Trees and Shrubs. Second Edition. Insects Feed Trees Shrubs. 1991; 560.

164. Speers CF. The pine spittle bug (*Aphrophora parallela* Say), Technical Publication No. 54. Bull N Y State Coll For. 1941;14: 1–65.

165. Yurtsever S. Records of spittle-producing insects (Hom., Cercopidae) in northwestern Turkey. Entomol Mon Mag. 2001;137: 77–78.

166. Biedermann R. Aggregation and survival of *Neophilaenus albipennis* (Hemiptera: Cercopidae) spittlebug nymphs. Eur J Entomol. 2003;100: 493–500.

167. Prestidge RA. Preliminary observations on the grassland leafhopper fauna of the central North Island Volcanic Plateau. N Z Entomol. 1989;12: 54–57. doi:10.1080/00779962.1989.9722564

168. Karahalıl B. Dorsal Colour/Pattern Phenotype Frequency of *Philaenus spumarius* (L.) in Some Turkish Populations and Host Plant Relations in Related Cercopidae Species. MS thesis, Trakya University. 2010.

169. Karadjova O, Krusteva H. Species composition and population dynamics of the harmful insect fauna (Hemiptera: Cicadomorpha, Fulgoromorpha and Sternorrhyncha) of winter triticale. Bulg J Agric Sci. 2017;22: 619–236.

170. Maneva V, Atanasova D, Nedelcheva T. Phytosanitary status and yield of kamut (*Triticum turgidum polonicum* L.) grown in organic and biodynamic farming. Agric Sci Technol. 2017;9: 42–44.

171. Garneau A. Liste de cicadaires récoltés en 1983 sur la rhubarb (*Rheum rhaponticum* L.) à Granby, Comté de Shefford. Fabreries. 1984;10: 77–79.

172. Beckett KIS, Robertson AB, Matthews PGD. Studies on gas exchange in the meadow spittlebug, *Philaenus spumarius*: the metabolic cost of feeding on, and living in, xylem sap. J Exp Biol. 2019;222: jeb191973. doi:10.1242/jeb.191973

173. Kwon O. Population dynamics of insects associated with *Rumex obtusifolius* in different habitats. Entomol Res. 2006;36: 73–78.

174. Winterbourn MJ. The arthropod fauna of bracken (*Pteridium aquilinum*) on the Port Hills, South Island, New Zealand. N Z Entomol. 1987;10: 99–104. doi:10.1080/00779962.1987.9722514

175. Lawton JH. The structure of the arthropod community on bracken. Bot J Linn Soc. 1976;73: 187–216.

176. Halarewicz A. Fulgoromorpha and Cicadomorpha (Hemiptera) infesting bracken (*Pteridium aquilinum*). Pol J Entomol. 2011;80: 451–456. doi:10.2478/v10200-011-0032-1

177. Yoder MV, Skinner LC, Ragsdale DW. Common buckthorn, *Rhamnus cathartica* L.: Available feeding niches and the importance of controlling this invasive woody perennial in North America. Proceedings of the XII International Symposium on Biological Control of Weeds, La Grande Motte, France, 22-27 April, 2007. CABI; 2008. pp. 232–237. doi:10.1079/9781845935061.0232

178. Osborn H. Meadow and Pasture Insects. Columbus, Ohio: The Educators' Press; 1939.

179. Pollard E. Hedges. ii. The effect of removal of the bottom flora of a hawthorn hedgerow on the fauna of the hawthorn. J Appl Ecol. 1968;5: 109–123. doi:10.2307/2401277

180. Cole DH, Ashman T-L. Sexes show differential tolerance to spittlebug damage and consequences of damage for multi-species interactions. Am J Bot. 2005;92: 1708–1713. doi:10.3732/ajb.92.10.1708

181. Crews LJ, McCully ME, Canny MJ, Huang CX, Ling LEC. Xylem feeding by spittlebug nymphs: some observations by optical and cryo-scanning electron microscopy. Am J Bot. 1998;85: 449–460. doi:10.2307/2446427

182. Jenser G, Hegab AM. On the distribution of *Philaenus spumarius* (Linné) imagoes on fruit-trees and the undergrowth (Homoptera). Folia Entomol Hung. 32: 231–233.

183. Cleaveland ML, Hamilton DW. The insect fauna of apple trees in southern Indiana, 1956 and 1957. Proc Indiana Acad Sci. 1958;68: 205–217.

184. Bleicher K, Orosz A, Cross J, Markó V. Survey of leafhoppers, planthoppers and froghoppers (Auchenorrhyncha) in apple orchards in South-East England. Acta Phytopathol Entomol Hung. 2010;45: 93–105. doi:10.1556/aphyt.45.2010.1.7

185. Batra SWT. Insects associated with weeds in the northeastern United States. ii. Cinquefoils, *Potentilla norvegica* and *P. recta* (Rosaceae). J N Y Entomol Soc. 1979;87: 216–222.

186. Anonymous. Spectacular concentration. Can Insect Pest Rev. 1945;23: 218–219.

187. Phillips JHH. An annotated list of Hemiptera inhabiting sour cherry orchards in the Niagara Peninsula, Ontario. Can Entomol. 1951;83: 194–205. doi:10.4039/Ent83194-8

188. Hasbroucq S, Casarin N, Czwienczek E, Bragard C, Grégoire J-C. Distribution, adult phenology and life history traits of potential insect vectors of *Xylella fastidiosa* in Belgium. Belg J Entomol. 2020;92: 1.

189. Osborn H. Homoptera in the Vicinity of Cranberry Lake. Papers from the Department of Forest Entomology, Technical Publication No 16. New York State College of Forestry at Syracuse University; 1922. pp. 24–54.

190. Angelova R, Gjonov I. Spittlebugs (Hemiptera: Cercopoidea) of Sarnena Sredna Gora (Bulgaria). ZooNotes. 2022.

191. Massee AM. The Hemiptera–Homoptera (Auchenorhyncha) associated with cultivated fruits. J Soceity Br Entomol. 1941;2: 99–109.

192. Batra SWT. Phytophages and Pollinators of *Galium* (Rubiaceae) in Eurasia and North America. Environ Entomol. 1984;13: 1113–1124. doi:10.1093/ee/13.4.1113

193. Schnaider Z. Feeding of *Aphrophora* spp. in osier beds, and control trials. Sylwan. 1960;104: 41–5.

194. Owen DF, Whiteway WR. *Buddleia davidii* in Britain: History and development of an associated fauna. Biol Conserv. 1980;17: 149–155. doi:10.1016/0006-3207(80)90045-2

195. Lambillion L-J. La cicadelle ecumeuse (*Aphrophora spumaria* Linne). Rev Mens Soc Entomol Namuroise. 1914; 71–73.

196. Patch EM. Aroostook potato insects. J Econ Entomol. 1922;15: 372–373.

197. Girsova NV, Bottner-Parker KD, Bogoutdinov DZ, Meshkov YI, Mozhaeva KA, Kastalyeva TB, et al. Diverse phytoplasmas associated with potato stolbur and other related potato diseases in Russia. Eur J Plant Pathol. 2016;145: 139–153. doi:10.1007/s10658-015-0824-3

198. Carle P, Moutous, Gilberte. Observations sur le mode de nutrition sur vigne de quatre espèces de cicadelles. Ann Epiphyt. 1965;16: 333–354.

199. Krstić O, Radonjić S, Hrnčić S, Cvrković T, Mitrović M, Kosovac A, et al. Diversity of the Auchenorrhyncha fauna in vineyards of Montenegro. Zašt Bilja. 2012;63: 108–113.

200. Rodrigues I, Rebelo MT, Baptista P, Pereira JA. Cicadomorpha community (Hemiptera: Auchenorrhyncha) in Portuguese vineyards with notes of potential vectors of *Xylella fastidiosa*. Insects. 2023;14: 251. doi:10.3390/insects14030251

201. Warren LO, Tadic M. The Fall Webworm, *Hyphantria cunea* (Drury). Bulletin 759. University of Arkansas, Agricultural Experiment Station, Fayetteville; 1970.

202. Brockerhoff EG, Suckling DM, Ecroyd CE, Wagstaff SJ, Raabe MC, Dowell RV, et al. Worldwide host plants of the highly polyphagous, invasive *Epiphyas postvittana* (Lepidoptera: Tortricidae). J Econ Entomol. 2011;104: 1514–1524. doi:10.1603/EC11160

203. Young OP. Host plants of the tarnished plant bug, *Lygus lineolaris* (Heteroptera: Miridae). Ann Entomol Soc Am. 1986;79: 747–762. doi:10.1093/aesa/79.4.747

204. Loxdale HD, Balog A, Harvey JA. Generalism in nature…the great misnomer: Aphids and wasp parasitoids as examples. Insects. 2019;10: 314. doi:10.3390/insects10100314

205. Pearce SL, Clarke DF, East PD, Elfekih S, Gordon KHJ, Jermiin LS, et al. Genomic innovations, transcriptional plasticity and gene loss underlying the evolution and divergence of two highly polyphagous and invasive *Helicoverpa* pest species. BMC Biol. 2017;15: 63. doi:10.1186/s12915-017-0402-6

206. Leonard DE. Bioecology of the gypsy moth. The Gypsy Moth: Research Toward Integrated Pest Management Forest Service Technical Bulletin 1584. Washington, D. C.: U. S. U.S. Department of Agriculture; 1981. pp. 9–29.

207. Ballesteros Mejia L, Arnal P, Hallwachs W, Haxaire J, Janzen D, Kitching IJ, et al. A global food plant dataset for wild silkmoths and hawkmoths and its use in documenting polyphagy of their caterpillars (Lepidoptera: Bombycoidea: Saturniidae, Sphingidae). Biodivers Data J. 2020;8: e60027. doi:10.3897/BDJ.8.e60027

208. Barringer L, Ciafré C. Worldwide feeding host plants of spotted lanternfly, with significant additions from North America. Environ Entomol. 2020;49. doi:10.1093/ee/nvaa093

209. Normark BB, Johnson NA. Niche explosion. Genetica. 2011;139: 551–564. doi:10.1007/s10709-010-9513-5

210. Grbić M, Van Leeuwen T, Clark RM, Rombauts S, Rouzé P, Grbić V, et al. The genome of *Tetranychus urticae* reveals herbivorous pest adaptations. Nature. 2011;479: 487–492. doi:10.1038/nature10640

211. Ossiannilsson F. The Auchenorrhyncha (Homoptera) of Fennoscandia and Denmark. Part 2: the families Cicadidae, Cercopidae, Membracidae, and Cicadellidae (excl. Deltocephalinae). Fauna Entomol Scand. 1981;7: 223–593.

212. Chartois M, Mesmin X, Quiquerez I, Borgomano S, Farigoule P, Pierre É, et al. Environmental factors driving the abundance of *Philaenus spumarius* in mesomediterranean habitats of Corsica (France). Sci Rep. 2023;13: 1901. doi:10.1038/s41598-023-28601-4

213. Halkka O, Mikkola E. The selection regime of *Philaenus spumarius* (L.) (Homoptera). In: Christiansen FB, Fenchel TM, editors. Measuring Selection in Natural Populations. Berlin, Heidelberg: Springer; 1977. pp. 445–463.

214. Thompson V. Spittlebug indicators of nitrogen-fixing plants. Ecol Entomol. 1994;19: 391–398. doi:10.1111/j.1365-2311.1994.tb00257.x

215. Hamilton KGA. The Spittlebugs of Canada. Homoptera: Cercopidae. The Insects and Arachnids of Canada. Part 10. Publication 1740. Ottawa: Agriculture Canada; 1982.

216. Thorne M, Wilson S, Wright M, Peck D. Twolined Spittlebug Identification Key. College of Tropical Agriculture and Human Resources, University of Hawaiʻi at Manoa; 2022.

217. Ashcroft T, George S. New to New Zealand: *Bathyllus albicinctus* (Hemiptera: Aphrophoridae) Whangarei. Initial Investigation & Technical Report/Entomology. 7 pp. New Zealand Ministry of Agriculture and Forestry; 2004.

218. Rösch V, Marques E, Miralles-Núñez A, Zahniser JN, Wilson MR. *Draeculacephala robinsoni* Hamilton, 1967 (Hemiptera: Auchenorrhyncha: Cicadellidae), a newly introduced species and genus in Europe with comments on its identification. Zootaxa. 2022;5116: 439–448. doi:10.11646/zootaxa.5116.3.8

219. Sergel R. Area expansion of the imported Nearctic cicadelline leafhopper *Graphocephala fennahi* Young, 1977 in Western Europe (Homoptera: Auchenorrhyncha). Articulata. 1987;3: 21–22.

220. Hill KBR, Marshall DC, Cooley JR. Crossing Cook Strait: Possible human transportation and establishment of two New Zealand cicadas from North Island to South Island (*Kikihia scutellaris* and *K. ochrina*, Hemiptera: Cicadidae). N Z Entomol. 2005;28: 71–80. doi:10.1080/00779962.2005.9722688

221. Maryańska-Nadachowska A, Kuznetsova VG, Lachowska D, Drosopoulos S. Mediterranean species of the spittlebug genus *Philaenus*: Modes of chromosome evolution. J Insect Sci. 2012;12.

222. Drosopoulos S, Maryańska-Nadachowska A, Kuznetsova VG. The Mediterranean: Area of origin of polymorphism and speciation in the spittlebug *Philaenus* (Hemiptera, Aphrophoridae). Zoosystematics Evol. 2010;86: 125–128. doi:10.1002/zoos.200900017

223. Boukhris-Bouhachem S, Souissi R, Abou Kubaa R, El Moujabber M, Gnezdilov V. Aphrophoridae as potential vectors of *Xylella fastidiosa* in Tunisia. Insects. 2023;14: 119. doi:10.3390/insects14020119

224. Drosopoulos S, Quartau JA. The spittle bug *Philaenus tesselatus* Melichar, 1899 (Hemiptera, Auchenorrhyncha, Cercopidae) is a distinct species. Zootaxa. 2002;68: 1–8. doi:10.11646/zootaxa.68.1.1

225. Maryańska-Nadachowska A, Drosopoulos S, Lachowska D, Kajtoch Ł, Kuznetsova VG. Molecular phylogeny of the Mediterranean species of *Philaenus* (Hemiptera: Auchenorrhyncha: Aphrophoridae) using mitochondrial and nuclear DNA sequences. Syst Entomol. 2010;35: 318–328.

226. Loxdale HD, Harvey JA. The ‘generalism’ debate: misinterpreting the term in the empirical literature focusing on dietary breadth in insects. Biol J Linn Soc. 2016;119: 265–282. doi:10.1111/bij.12816

227. Biello R, Mathers TC, Mugford ST, Liu Q, Rodrigues ASB, Neto AC, et al. Draft genome assembly version 1 of the meadow spittlebug *Philaenus spumarius* (Linnaeus, 1758) (Hemiptera, Aphrophoridae). Zenodo; 2020. doi:10.5281/zenodo.3368385

228. Thompson V. Insect-plant-fungus interactions in mycorrhizal associations, with a focus on spittlebugs and ectomycorrhizal host plants. Ecol Entomol. 2022;47: 915–929. doi:10.1111/een.13192

229. Singer MS. Evolutionary ecology of polyphagy. K. Tilmon, (ed.). Specialization, Speciation, and Radiation–Evolutionary Biology of Herbivorous Insects. Berkeley: University of California Press; 2008. pp. 29–42.

230. Jaenike J. Host specialization in phytophagous insects. Annu Rev Ecol Syst. 1990;21: 243–273. doi:10.1146/annurev.es.21.110190.001331

231. Forister ML, Novotny V, Panorska AK, Baje L, Basset Y, Butterill PT, et al. The global distribution of diet breadth in insect herbivores. Proc Natl Acad Sci. 2015;112: 442–447. doi:10.1073/pnas.1423042112

232. Formisano G, Iodice L, Cascone P, Sacco A, Quarto R, Cavalieri V, et al. *Wolbachia* infection and genetic diversity of Italian populations of *Philaenus spumarius*, the main vector of *Xylella fastidiosa* in Europe. PLOS ONE. 2022;17: e0272028. doi:10.1371/journal.pone.0272028

233. Maryańska-Nadachowskaa A, Sanaieb E, Kajtocha Ł. High genetic diversity in southwest Asian populations of *Philaenus spumarius* (Hemiptera: Auchenorrhyncha). Zool Middle East. 2015;61: 264–272. doi:10.1080/09397140.2015.1069243

234. Lester K, Murphy K, McCluskey A, Cairns R, Fraser K, Kenyon D. *Xylella fastidiosa*: an overview of research at SASA. The Dundee Conference, Crop Production in Northern Britain, Dundee, UK, 25-26 February 2020. The Association for Crop Protection in Northern Britain; 2020. pp. 45–50.

235. Hoffman GD, McEvoy PB. The mechanism of trichome resistance in *Anaphalis margaritacea* to the meadow spittlebug *Philaenus spumarius*. Entomol Exp Appl. 1985a;39: 123–129. doi:10.1111/j.1570-7458.1985.tb03552.x

236. Wheeler AG. Phytophagous arthropod fauna of crownvetch in Pennsylvania. Can Entomol. 1974;106: 897–908. doi:10.4039/Ent106897-9

237. Morente M, Ramírez M, Lago C, de las Heras-Bravo D, Benito A, Moreno A, et al. Habitat manipulation for sustainable management of *Philaenus spumarius*, the main vector of *Xylella fastidiosa* in Europe. Pest Manag Sci. 2022;78: 4183–4194.

238. Wheeler AG. *Lepyronia coleoptrata* (Homoptera: Cercopidae), an immigrant spittlebug in North America : Distribution, seasonal history, and host plants. Proc Entomol Soc Wash. 1991;93: 463–470.

239. Press MC, Whittaker JB. Exploitation of the xylem stream by parasitic organisms. Philos Trans R Soc Lond B Biol Sci. 1993;341: 101–111.

240. Purcell AH, Finlay A. Evidence for noncirculative transmission of Pierce’s Disease bacterium by sharpshooter leafhoppers. Phytopathology. 1979;69: 393–395. doi:10.1094/Phyto-69-393

241. Avosani S, Nieri R, Mazzoni V, Anfora G, Hamouche Z, Zippari C, et al. Intruding into a conversation: How behavioral manipulation could support management of *Xylella fastidiosa* and its insect vectors. J Pest Sci. 2023 [cited 27 May 2023]. doi:10.1007/s10340-023-01631-7

242. Avosani S, Daher E, Franceschi P, Ciolli M, Verrastro V, Mazzoni V. Vibrational communication and mating behavior of the meadow spittlebug *Philaenus spumarius*. Entomol Gen. 2020;40: 351–363. doi:10.1127/entomologia/2020/0983

243. Cruaud A, Gonzalez A-A, Godefroid M, Nidelet S, Streito J-C, Thuillier J-M, et al. Using insects to detect, monitor and predict the distribution of *Xylella fastidiosa*: a case study in Corsica. Sci Rep. 2018;8: 15628. doi:10.1038/s41598-018-33957-z

244. Pocock MJO, Tweddle JC, Savage J, Robinson LD, Roy HE. The diversity and evolution of ecological and environmental citizen science. PLOS ONE. 2017;12: e0172579. doi:10.1371/journal.pone.0172579

245. Miller-Rushing A, Primack R, Bonney R. The history of public participation in ecological research. Front Ecol Environ. 2012;10: 285–290. doi:10.1890/110278

246. Lewandowski E, Specht H. Influence of volunteer and project characteristics on data quality of biological surveys. Conserv Biol. 2015;29: 713–723. doi:10.1111/cobi.12481

247. Poisson AC, McCullough IM, Cheruvelil KS, Elliott KC, Latimore JA, Soranno PA. Quantifying the contribution of citizen science to broad-scale ecological databases. Front Ecol Environ. 2020;18: 19–26. doi:10.1002/fee.2128

248. Sumner S, Bevan P, Hart AG, Isaac NJB. Mapping species distributions in 2 weeks using citizen science. Insect Conserv Divers. 2019;12: 382–388. doi:10.1111/icad.12345

 Click here to access/download;Figure;Fig 1.tif

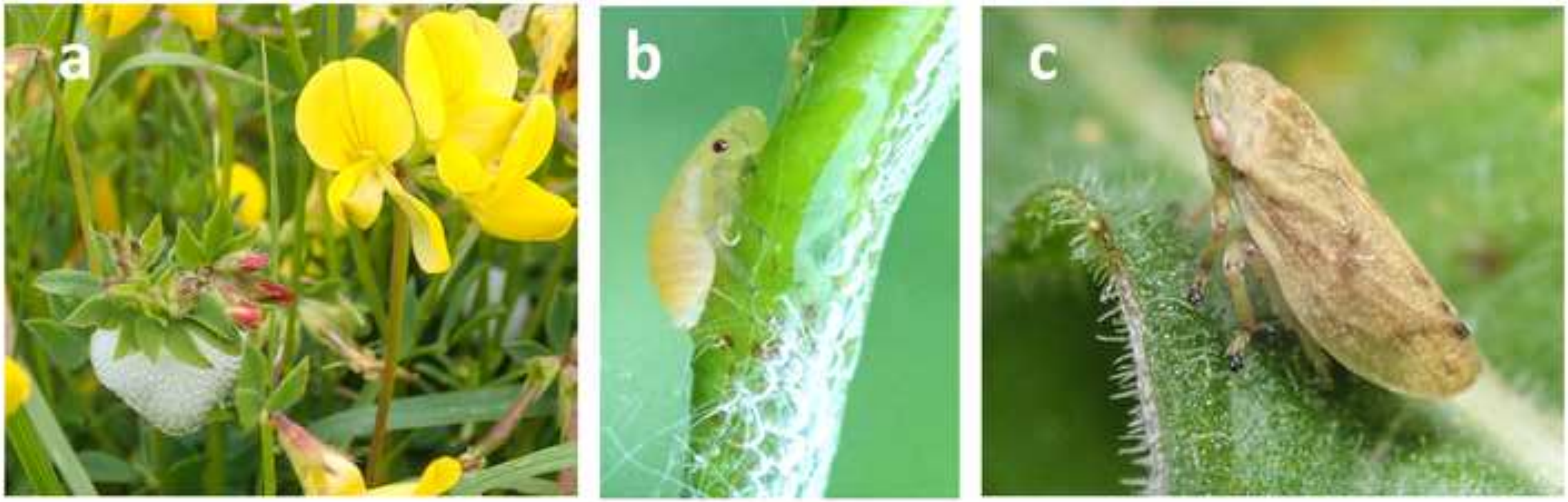

Supplement: Attachment — Submitted filename: PONE-D-23-20831_reviewer.pdf [file pone.0291734.s001.pdf]
