# Supplementary material for: The most polyphagous insect herbivore? Host plant associations of the Meadow spittlebug, Philaenus spumarius (L.)
Source: PLoS One. 2023 Oct 4;18(10):e0291734. doi: 10.1371/journal.pone.0291734 (PMC10602594; doi:10.1371/journal.pone.0291734)
Supplement: Attachment — Submitted filename: Response to Reviewers.docx [file pone.0291734.s002.docx]

Dr. Janice L. Bossart
Academic Editor
PLOS ONE

Dear Dr Bossart

Thank you for considering our submission to PLOS ONE and for the perceptive comments and constructive suggestions for improvement of the manuscript. Our responses to these comments are listed below. We have reproduced in bold the comments from the journal; our response to each one then follows in normal font. Line numbers in our responses refer to the revised manuscript without track changes.

**Journal Requirements:**

1. **Journal style requirements**

We have reviewed the style requirements and are satisfied that the manuscript meets them.

2. **Data Availability statement**

Our entire dataset is contained within Table 1.

3. **Reference list**

We have reviewed the reference list and are satisfied that it is complete, correct and meets the formatting requirements.

**Additional Editor Comments:**

**Both reviewers feel your manuscript will have significant impact in the scientific community, especially for researchers dealing with Xylella fastidiosa impacts. There concerns were generally minor and shouldn't require much time to address.**

Thank you for this endorsement of our work.

**Comments to the Author**

**Reviewer #1: The manuscript is a comprehensive review of the meadow spittlebug host plants. I have just minor comments and suggestions the authors will find in the attached pdf.**
Thank you for this assessment.

**In addition:
1) I think it'd be better to present table 1 as supplementary material**
We note the reviewer’s suggestion, but we feel strongly that Table 1 belongs, as currently positioned, in the main body of the paper. Our work is unusual, in that its main purpose is to bring directly to the scientific community an annotated, complete compilation of *Philaenus spumarius* host plants. The primary target audience is the large and growing contingent of scientists and agronomists working with the plant pathogen *Xylella fastidiosa*. The full table of *P. spumarius* hosts will become a staple of their work and they will consult it frequently, most often searching for host plants that they have observed in association with *P. spumarius* in natural or agricultural habitats where *P. spumarius* is a vector or potential vector. Their needs will be best served by having the table visible, readily accessible and at hand, with no need to call up supplementary materials separate from the pdf or html versions of the primary work. We note that the European Food Safety Authority (EFSA) publishes a comparable compilation of *X. fastidiosa* host plants. The tables for their compilation of several hundred *Xylella* host plant species are published in full in the main body of the text and heavily consulted by workers in *Xylella*-related studies.

We understand that the table is long and would be impractical to print as part of a traditional print-based journal article. That was a primary motivation for submitting to PLOS ONE. In a solely on-line venue, issues of page length are not a barrier to presenting detailed data sets in full where they are central to the story and to the interests of the target audience. For our readers, we would like to take full advantage of this flexibility and present the core of our work, and the part of it most likely to be accessed regularly, in the most visible and accessible way.

**2) I suggest to remove the conclusions on the importance of citizen science in surveys on Xylella fastidiosa vectors. The bacterium transmission dynamic is indeed far more complicated than the "simple" assessment of the number of spittles in a certain area/orchard, and these kind of statements could be easily misinterpreted by both the scientific community and the stakeholders.**

We completely agree that the number of spittles is only one component in assessing Xylella transmission risk. However, this was only two sentences at the end of this section and we feel that it would be useful to retain the rest of the text, even though the reviewer has suggested deleting the whole section. Accordingly, we have modified the final two sentences as follows:

*At least in the UK, therefore, spittle on herbaceous dicots could be used for rapid assessment of both the presence and relative abundance of* P. spumarius*. Such estimates could form part of a future surveillance strategy for* X. fastidiosa*, although many other factors would need to be considered when assessing the risk of bacterial transmission.*

**Reviewer #2: The authors carried out a quite impressive research on the host plant range of the spittlebug Philaenus spumarius. They both delved the, often ancient, literature but provided also the results of an interesting citizen science project on the topic embedded in the EU project BRIGIT. The results are interesting and useful, especially for the many scientists studying the insect as the key vector of Xylella fastidiosa in Europe. The impressive list of host plants of the extreme polyphagous insect topic of this article surely may contain some mistakes or imprecisions, but it try to make order in a otherwise cluttered amount of literature and information, some really old and not easily accessible even to the average expert.**
Thank you for these positive comments.

**I have only few little corrections and considerations.**

**#1
The MS provides some tables but lacks graphs. There is not a graphical way to condense some of the information present in table 2 or 3 in a plot and maybe place the table as a supplementary material? It could be more immediate to the reader the importance of some plant family as host plants or the comparison between P. spumarius and other extreme polyphagous insects. This is just a suggestion to increase the readability of the paper and it is not compulsory.**

We have experimented with various graphical representations of these two tables but have concluded that the data are best portrayed in tabular format.

**l. 145-146: “with which VT is familiar”. I found this expression a little too personal and not really supported by data. It would be better to cite some literature on California climate or briefly describe which climate type the authors referred to. In any case this clarification is in my opinion not necessary and can be avoided.**

We accept the point about this expression. We contend, however, that some clarification of whether the observations made by DeLong & Severin referred to nymphs or adults is useful. Accordingly, we have deleted the sentence in question and replaced it with the following (lines 146-151):

*All were made in Alameda County, on the sunnier, warmer side of San Francisco Bay, in April or the first three weeks of May, or in San Francisco, on the foggier, cooler side of the bay, in April, May or the first week of July. In these areas, for the periods in question, most or all P. spumarius individuals are still in the nymphal stage (VT observations) and it may be reasonably inferred that DeLong & Severin observed nymphs for all listed hosts.*

**l. 305: “fantastic”. Although I agree that P. spumarius is a fantastic insect this expression is maybe a little too personal in my opinion.**

This is a fair point. We have replaced ‘fantastic’ with ‘extraordinary’ (line 308).

**Table 3: “weighted occurrence index”. Although the authors state that this is a rough way to account for differences in number of species per family, it would be ideal if the number of species/family would be weighted for the species per family present in the area of distribution of P. spumarius and not worldwide. Is it feasible to do so or is it too much work and beyond the scope of this MS? Think about that.**

Thank you for this interesting point. It is true that, ideally, the summation of plant species in each family should include only those that overlap with the actual distribution of *P. spumarius*. However, such an analysis would require extracting the geographical distributions of every species in the 117 plant families concerned. Apart from the work involved in such an exercise, the ranges of some of these may not be known with sufficient spatial precision to make this a worthwhile endeavour. Accordingly, we have inserted the following sentence into the text on lines 469-471:

*Given that* P. spumarius *has an essentially temperate distribution, however, it should be noted that the index will be highly conservative for plant families that have a large proportion of species in the tropics.*

**l. 347: I am not a English native speaker, and I had never read this expression before. Consider if it is really needed a similar colloquial English expression.**

This is a perfectly valid point. We have replaced the sentence with the following one (line 350):

*We start by comparing* P. spumarius *with other insects in terms of the number of host species exploited.*

**l. 417: delete one “of”.**

Done (line 450).

**l. 468: correct Rubiaciae in Rubiaceae.**

Done (line 473).

**l. 495-96: I have to say that I am a little skeptical about the reports on the apparent avoidance of Securigera varia by P. spumarius nymphs. I have personally found spittlebug nymphal stages (not many) on closely related species and I find difficult to think that this particular species is anything really special for P. spumarius. I just suggest to be more careful in presenting this plant as a possible candidate for suppressing the population of the insect pest, also because the study of Morente et al. cited used very different plants from other taxonomic families as trap crops. More dedicated studies are needed before planting a Fabaceae plant to suppress spittlebug population in a X. fastidiosa susceptible crop orchard!**

This is a valid point given the current lack of direct experimental evidence on the susceptibility of this plant species to spittlebug feeding. We have modified the text to be more cautious, as follows (lines 499-502):

*This suggests, subject to experimental verification, that there may be something exceptional about its biology that repels* P. spumarius*. If so, it might be a candidate species for understory plantings in orchards and groves where there is a desire to suppress* P. spumarius *vector populations [237].*

**l. 527: statement not really supported, we do not know well the host range of adults, given also the underlined difficulty in assessing a real host plant, i.e., a plant on which they feed. I would suggest to stress the lack of information on adult stage host plant range and not assume that it is comparable to the one of nymphal stages.**

This is an important point; thank you. We have modified the sentence as follows (lines 532-536):

*In contrast, adults are known to be effective vectors [110] but their exact host range is much less certain, with many fewer hosts documented (Table 1), this in turn substantially due to the difficulty noted in distinguishing between a functional host plant and one on which the insect is merely positioned.*

**Responses to in-text comments by Reviewer #1 (line numbers refer to PDF file with reviewer comments). Please note that all suggestions for minor single-word adjustments or typographic errors have been accepted.**

**L 21: Please summarize the main goal of the present work and the methodology**

To provide a broader context, we have inserted the following sentence at the start (lines 23-24):

*A comprehensive list of all known host plant species utilised by the Meadow Spittlebug* (Philaenus spumarius *(L.)*) *is presented, compiled from published and unpublished sources.*

**L 33-36: Unclear, please rephrase**

We assume the reviewer is referring to the word ‘captured’ (line 36). We have now replaced this with ‘recorded’ which we suggest clarifies the meaning.

**L 46-7: Remove, it's not consistent with the statements below (that there was a proliferation of studies on spittlebug hosts upon Xylella first outbreak)**

The key concept here is ‘comprehensive review’. We have re-phrased the next sentence as follows (replacement words underlined, lines 49-52):

*Concern with X. fastidiosa has led to a proliferation of recent studies adding new* P. spumarius *hosts (e.g. [6–11]), including the BRIGIT citizen scientist initiative in Britain that enlisted amateurs to identify* P. spumarius *host plants [12].*

**L 171: Please explain what do you mean by "problematic"**

We are referring to records where the species identification may be in question. We have replaced ‘problematic’ with ‘unreliable’.

**L 211-2: Please rephrase.**

We are unclear why this sentence is problematic. Nevertheless, we have added the word ‘representing’ to make the sentence clearer (line 216):

*We also recognize the possibility that some of the original plant identifications may have been in error, representing another, hopefully small, source of noise in the data.*

**Table 3: How can it be >1? (referring to the weighted occurrence index) This comment is repeated for lines 367-9.**

As explained in the table’s legend and on lines 472-3, the weighted occurrence index is a percentage and can therefore assume values greater than one.

**L 376: Please add also Avosani, S., Nieri, R., Mazzoni, V., Anfora, G., Hamouche, Z., Zippari, C., ... & Cornara, D. (2023). Intruding into a conversation: how behavioral manipulation could support management of Xylella fastidiosa and its insect vectors. Journal of Pest Science, 1-17.**

We have added this reference to the in-text citation (line 379).

Thank you once again for considering our paper for publication in PLOS ONE. We look forward to receiving your decision on the revised version.

Yours sincerely,

Alan Stewart

Claire Harkin

Vinton Thompson
